# Supplementary material for: Biocatalytic Synthesis of Coumarin S-Glycosides: Towards Non-Cytotoxic Probes for Biomedical Imaging and Sensing
Source: Molecules. 2024 Mar 16;29(6):1322. doi: 10.3390/molecules29061322 (PMC10974290; doi:10.3390/molecules29061322)
Supplement: Supplementary file 1 [file molecules-29-01322-s001.zip › molecules-2912421-supplementary.pdf]

Biocatalytic Synthesis of Coumarin *S*-Glycosides: Towards Non-Cytotoxic Probes for Biomedical Imaging and Sensing

Nastassja Burrini[1], Arnaud Pâris[1], Guillaume Collet[2], Pierre Lafite[1,*] and Richard Daniellou[2,3,*]

[1] Institut de Chimie Organique et Analytique, University of Orléans CNRS UMR7311, F-45067 Orléans, France; nastassja.burrini@univ-orleans.fr (N.B.)
[2] Chaire de Cosmétologie, AgroParisTech, 10 rue Léonard de Vinci, F-45100 Orléans, France
[3] Université Paris-Saclay, INRAE, AgroParisTech, Micalis Institute, F-78350 Jouy-en-Josas, France

* Correspondence: pierre.lafite@univ-orleans.fr (P.L.); richard.daniellou@agroparistech.fr (R.D.)

SUPPLEMENTAL INFORMATION

Figures S1 to S18: HPLC/UV chromatograms of substrates, reaction mixture, and purified **S-1** to **S-6** products

Figures S19 to S24 : HRMS spectra of purified **S-1** to **S-6**

Figures S25 to S27: ^{1}H and ^{13}C NMR of undescribed compounds **S-2, S-5** and **S-6.**

Figures S28 to S43 : Absorption spectra and emission and Excitation fluorescence spectra of compounds **4-MUB**, **7-MC** and products **S-1** to **S-6**

Figure S44: LOD and LOQ determination for compound **S-3**

HPLC Analysis of reactions and purified products

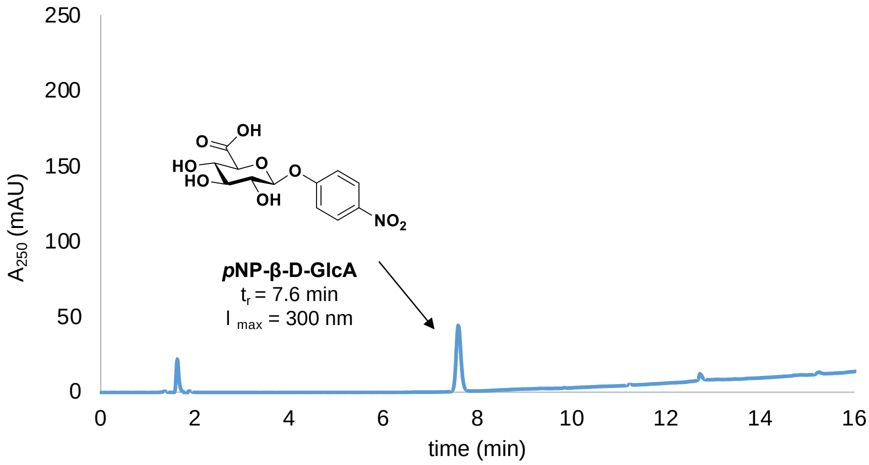

Figure S1. HPLC/UV (250 nm) chromatogram of sugar donor *p*NP-β-D-GlcA

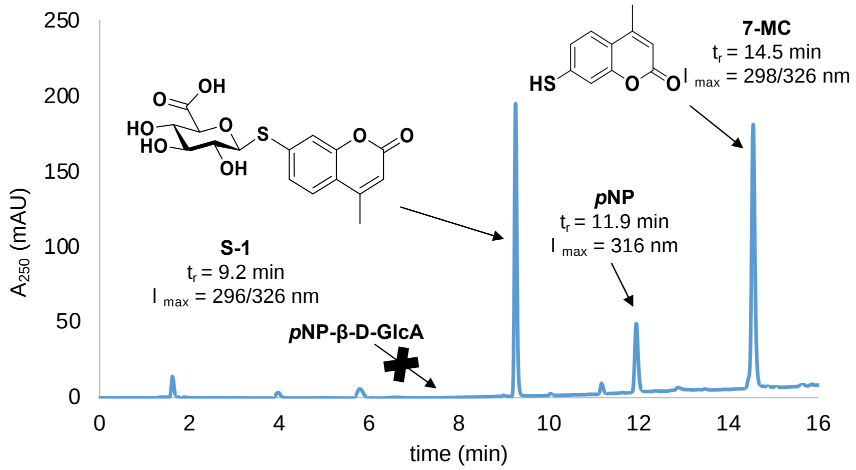

Figure S2. HPLC/UV (250 nm) chromatogram of thioglycoligation reaction for the synthesis of **S1** (24h, 37°C, donor:acceptor = 1:2.5)

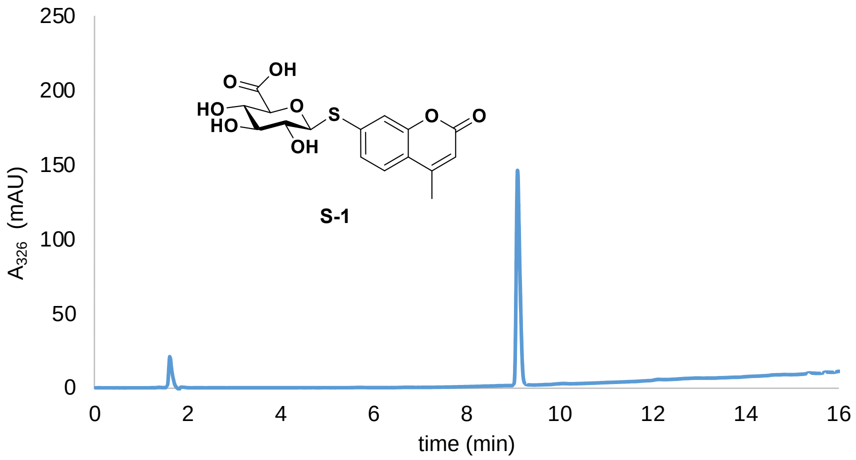

Figure S3. HPLC/UV (326 nm) chromatogram of purified S-glycoside **S-1**

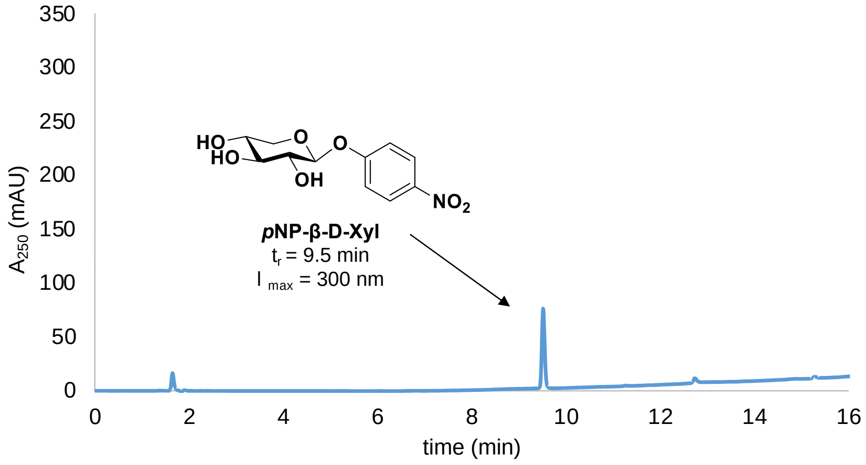

Figure S4. HPLC/UV (250 nm) chromatogram of sugar donor substrate *p*NP-β-D-Xyl

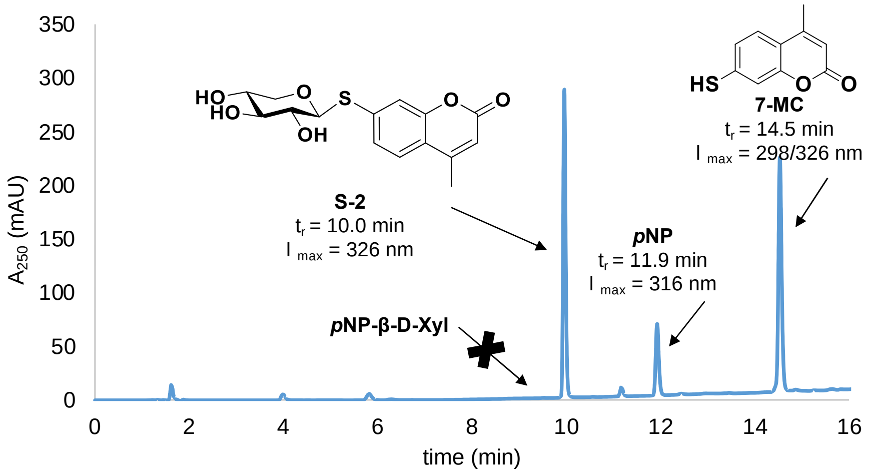

Figure S5. HPLC/UV (250 nm) chromatogram of thioglycoligation reaction for the synthesis of **S-2** (donor:acceptor = 1:2.5)

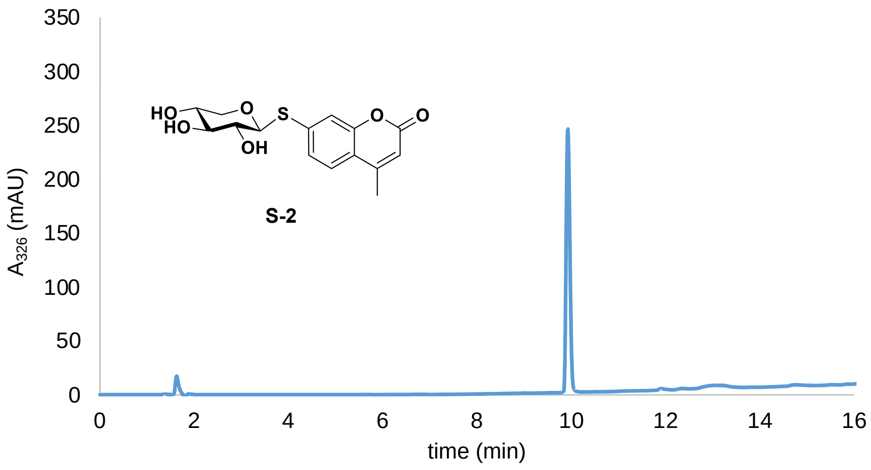

Figure S6. HPLC/UV (326 nm) chromatogram of purified S-glycoside **S-2**

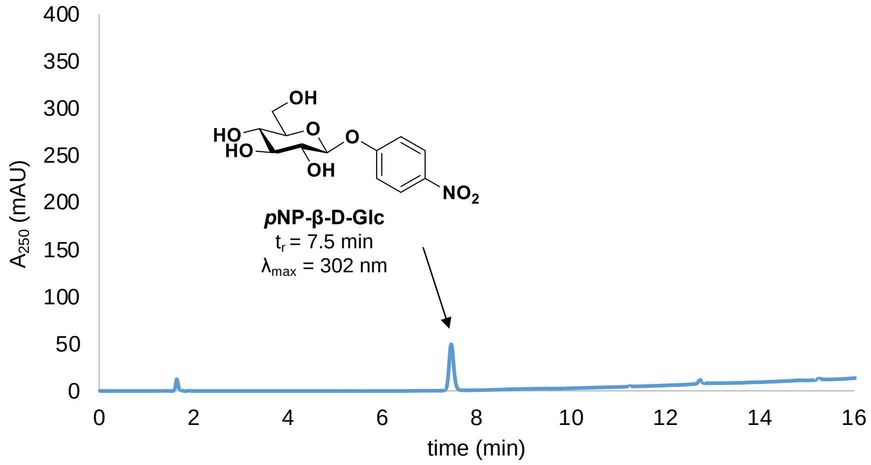

Figure S7. HPLC/UV (250 nm) chromatogram of sugar donor substrate *p*NP-β-D-Glc

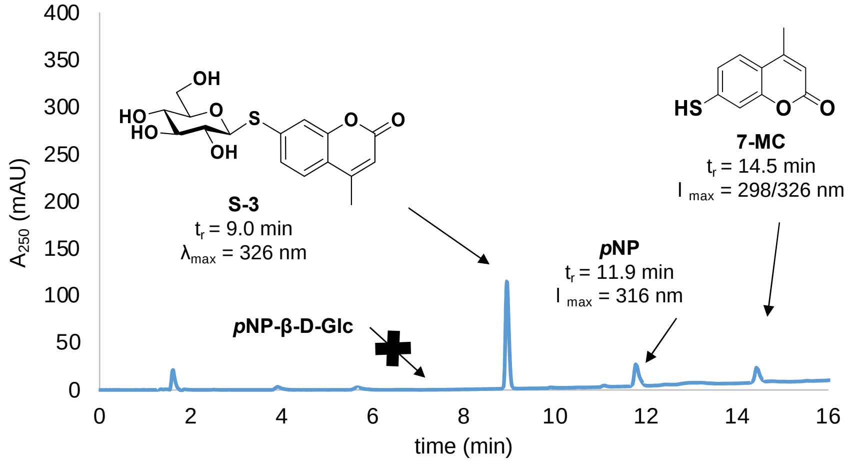

Figure S8. HPLC/UV (250 nm) chromatogram of thioglycoligation reaction for the synthesis of **S-3** (24h, 37°C, donor:acceptor = 1:2.5)

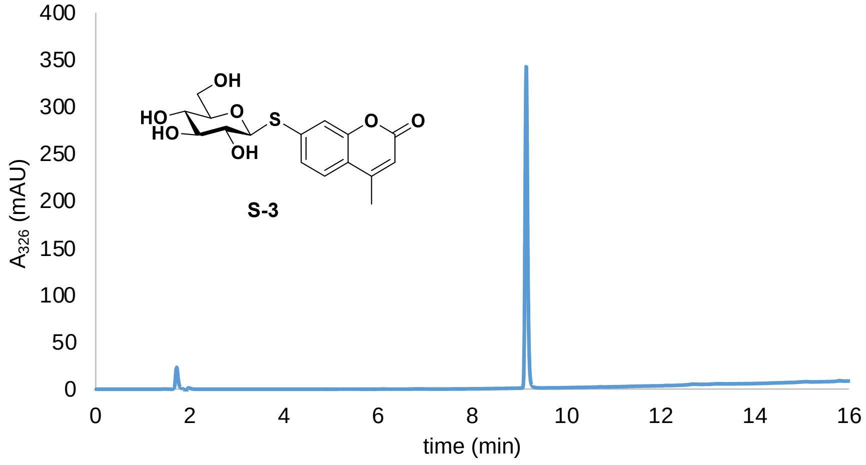

Figure S9. HPLC/UV (326 nm) chromatogram of purified S-glycoside **S-3**

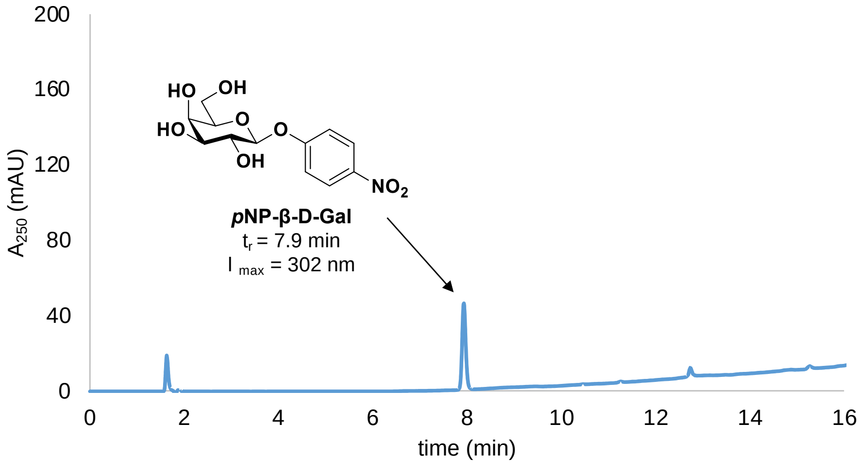

Figure S10. HPLC/UV (250 nm) chromatogram of sugar donor substrate *p*NP-β-D-Gal

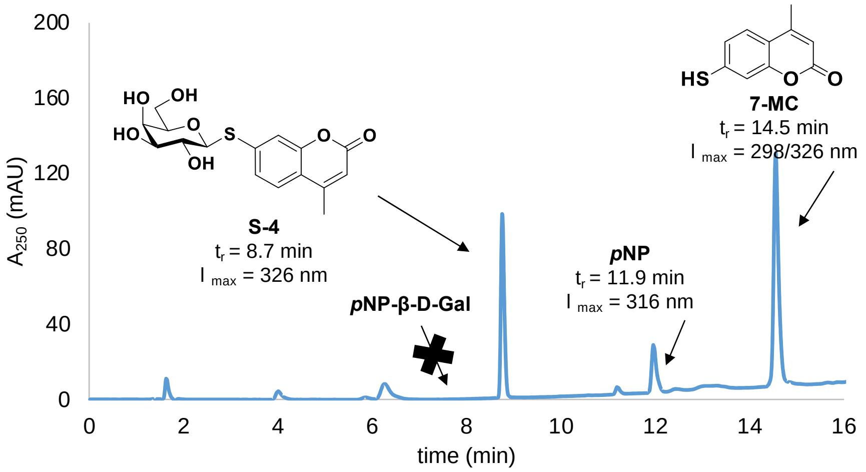

Figure S11. HPLC/UV (250 nm) chromatogram of thioglycoligation reaction for the synthesis of **S-4** (donor:acceptor = 1:2.5)

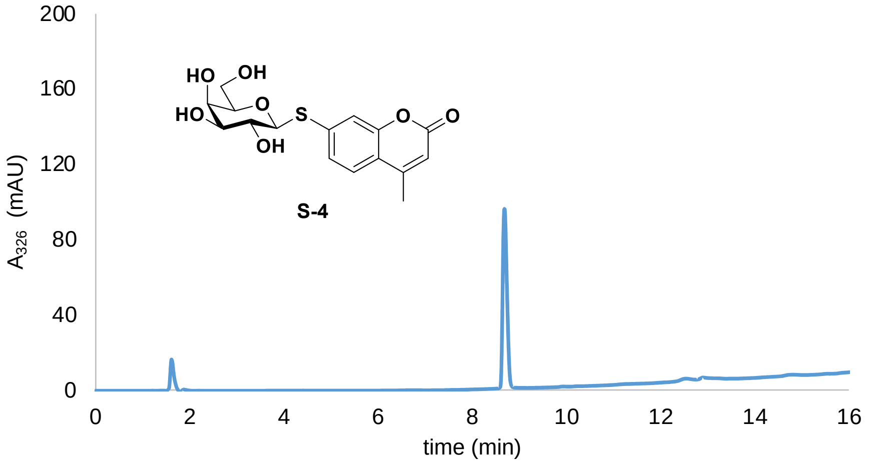

Figure S12. HPLC/UV (326 nm) chromatogram of purified S-glycoside **S-4**

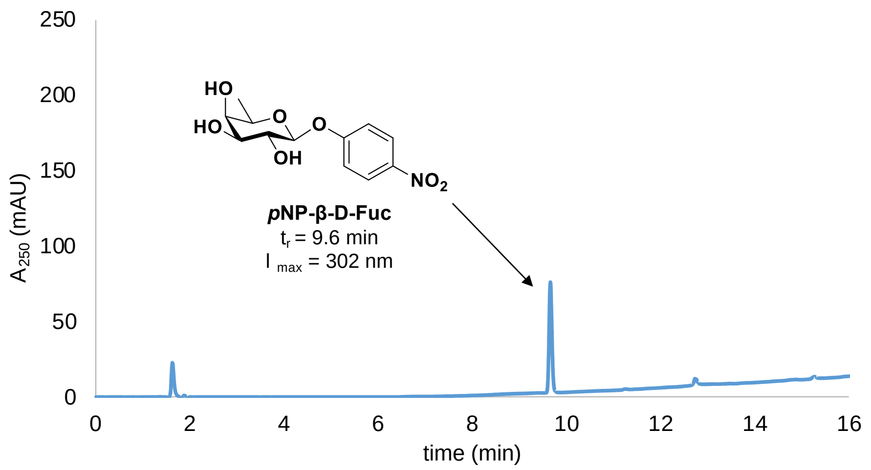

Figure S13. HPLC/UV (250 nm) chromatogram of sugar donor substrate *p*NP-β-D-Fuc

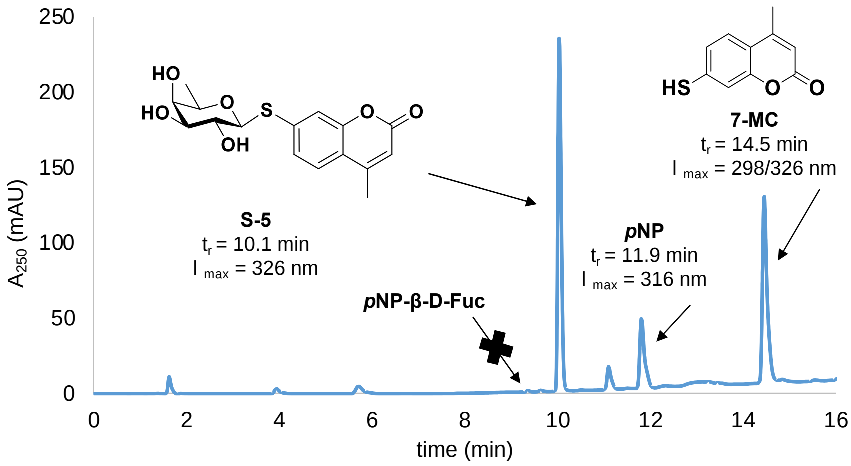

Figure S14. HPLC/UV (250 nm) chromatogram of thioglycoligation reaction for the synthesis of **S-5** (donor:acceptor = 1:2.5)

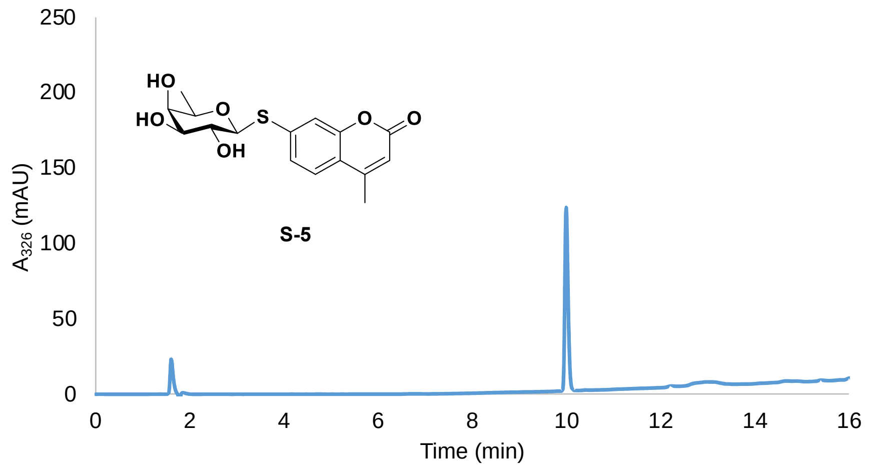

Figure S15. HPLC/UV (326 nm) chromatogram of purified S-glycoside **S-5**

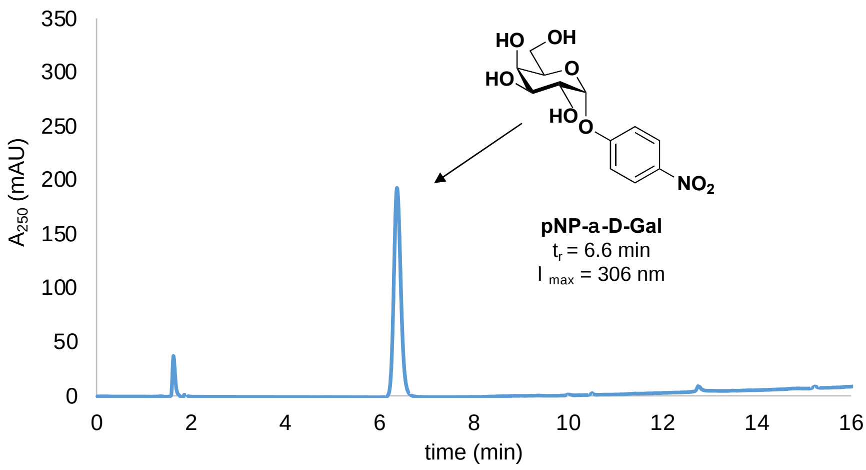

Figure S16. HPLC/UV (250 nm) chromatogram of sugar donor substrate *p*NP-α-D-Gal

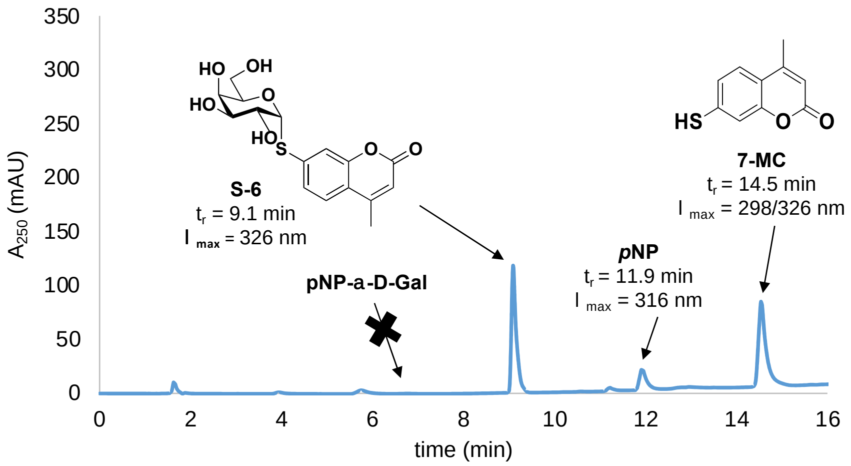

Figure S17. HPLC/UV (250 nm) chromatogram of thioglycoligation reaction for the synthesis of **S-6** (donor:acceptor = 1:2.5)

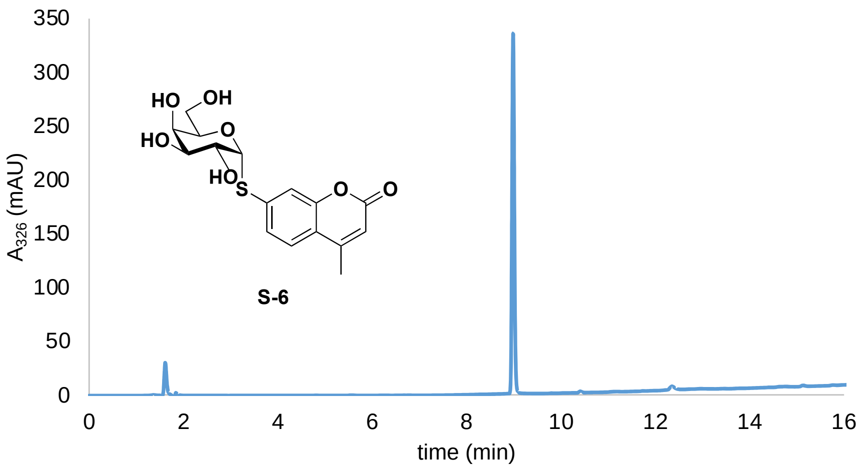

Figure S18. HPLC/UV (326 nm) chromatogram of purified **S-6**

HRMS Analysis of purified products

Synthèse et Analyse pour La Santé, l'Agronomie et bien-être
Spectrométrie de Masse Haute Résolution

**Analysis Info**		Acquisition Date	01/03/2023 11:22:55
Sample Name	**GlcA-Coumarin**	Instrument / Ser#	maXis 255552.00086
Analysis Name	X071598CYC.d	Method	positif-6.m

Acquisition Parameter

Source Type	ESI	Ion Polarity	Positive	Set Nebulizer	0.6 Bar
Scan Begin	50 m/z	Set Capillary	4500 V	Set Dry Heater	200 °C
Scan End	2500 m/z	Set Collision Cell RF	1800.0 Vpp	Set Dry Gas	7.0 l/min

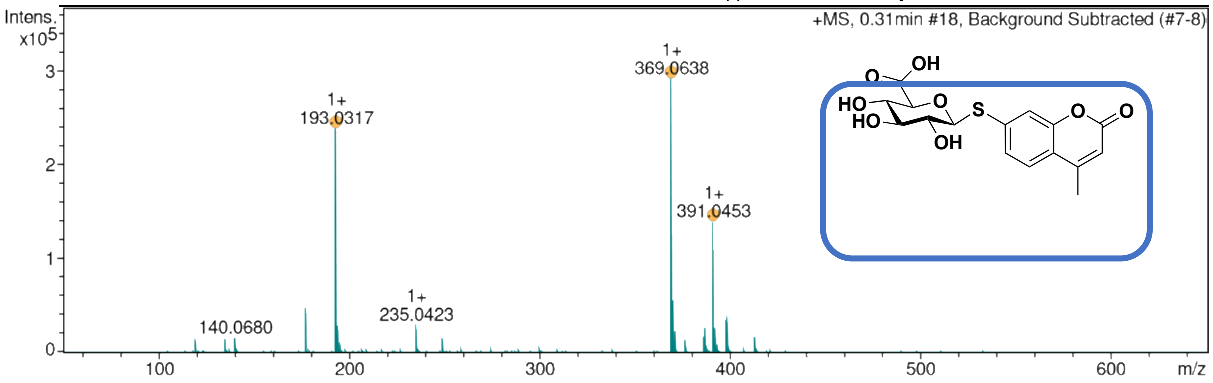

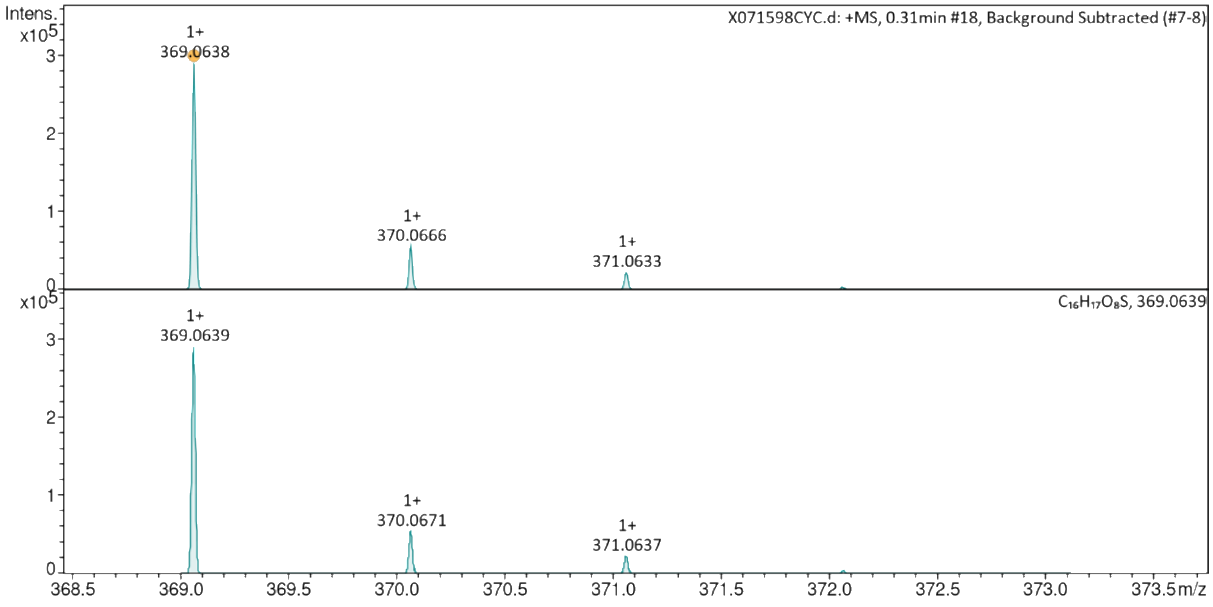

Meas. m/z	z	#	Ion Formula	m/z	err [ppm]	mSigma	rdb	e⁻ Conf
193.031669	1+	1	C10H9O2S	193.031777	0.6	4.1	7.0	even
369.063796	1+	1	C16H17O8S	369.063865	0.2	2.0	9.0	even
391.045312	1+	1	C16H16NaO8S	391.045809	1.3	6.3	9.0	even

Synthèse et Analyse pour La Santé, l'Agronomie et le bien-être
Spectrométrie de Masse Haute Résolution

**Analysis Info**		Acquisition Date	01/03/2023 22:18:57
Sample Name	**GlcA-Coumarin**	Instrument / Ser#	maXis 255552.00086
Analysis Name	X071599CYC.d	Method	Negatif.m

Acquisition Parameter

Source Type	ESI	Ion Polarity	Negative	Set Nebulizer	0.6 Bar
Scan Begin	50 m/z	Set Capillary	4000 V	Set Dry Heater	200 °C
Scan End	3000 m/z	Set Collision Cell RF	500.0 Vpp	Set Dry Gas	7.0 l/min

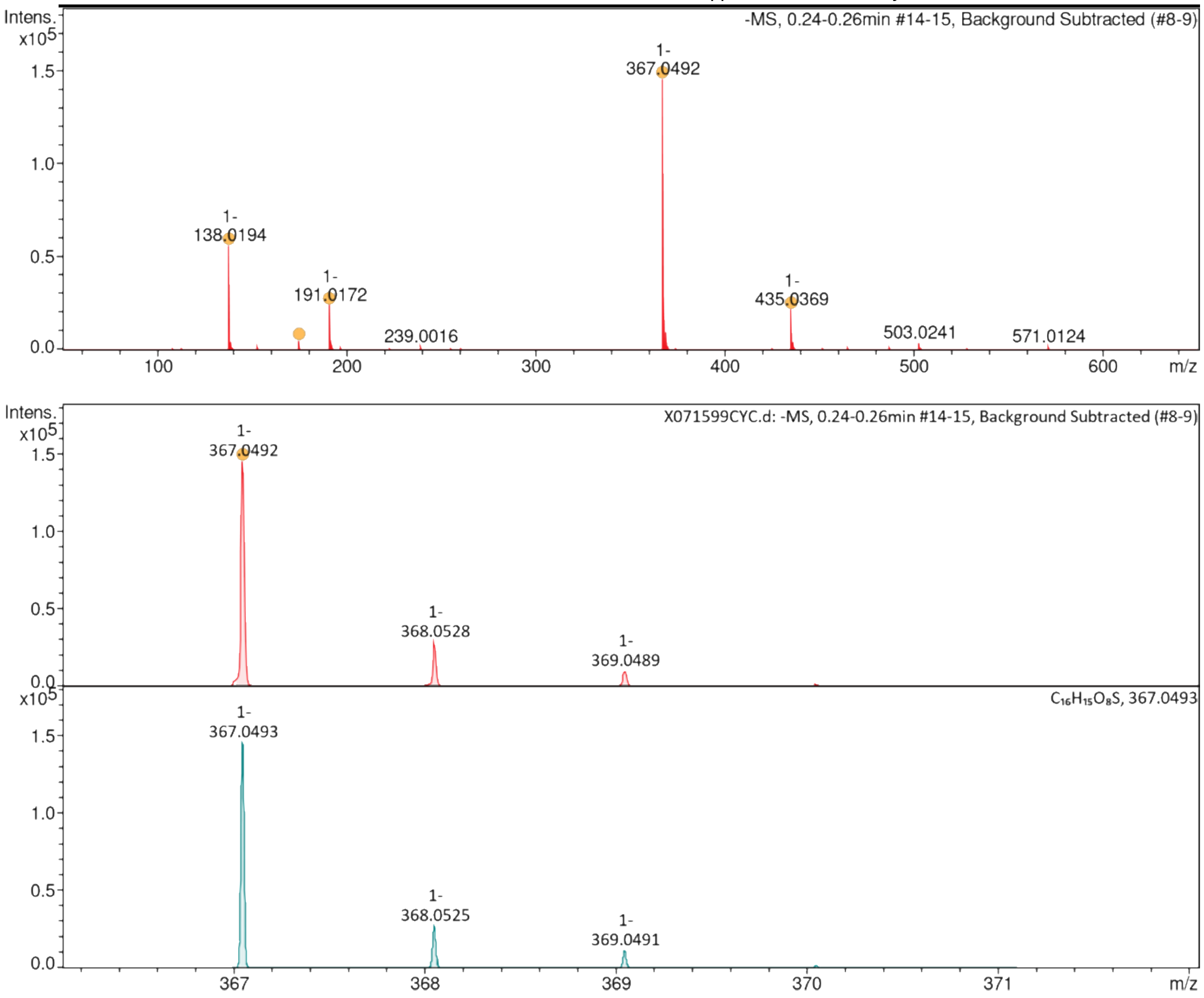

Meas. m/z	#	Ion Formula	m/z	err [ppm]	mSigma	rdb	e⁻ Conf	N-Rule
138.019357	1	C6H4NO3	138.019667	2.2	3.8	5.0	even	ok
175.025353	1	C6H7O6	175.024812	-3.1	n.a.	3.0	even	ok
191.017155	1	C10H7O2S	191.017224	0.4	7.3	7.0	even	ok
367.049228	1	C16H15O8S	367.049312	0.2	7.6	9.0	even	ok
435.036878	1	C17H16NaO10S	435.036736	-0.3	11.1	9.0	even	ok

Figure S19: MS spectra (positive and negative mode) of product **S-1**

Synthèse et Analyse pour La Santé, l'Agronomie btdn-être
Spectrométrie de Masse Haute Résolution

**Analysis Info**		Acquisition Date	01/03/2023 11:21:23
Sample Name	**Xyl-Coumarin**	Instrument / Ser#	maXis 255552.00086
Analysis Name	X071596CYC.d	Method	positif-6.m

Acquisition Parameter

Source Type	ESI	Ion Polarity	Positive	Set Nebulizer	0.6 Bar
Scan Begin	50 m/z	Set Capillary	4500 V	Set Dry Heater	200 °C
Scan End	2500 m/z	Set Collision Cell RF	1800.0 Vpp	Set Dry Gas	7.0 l/min

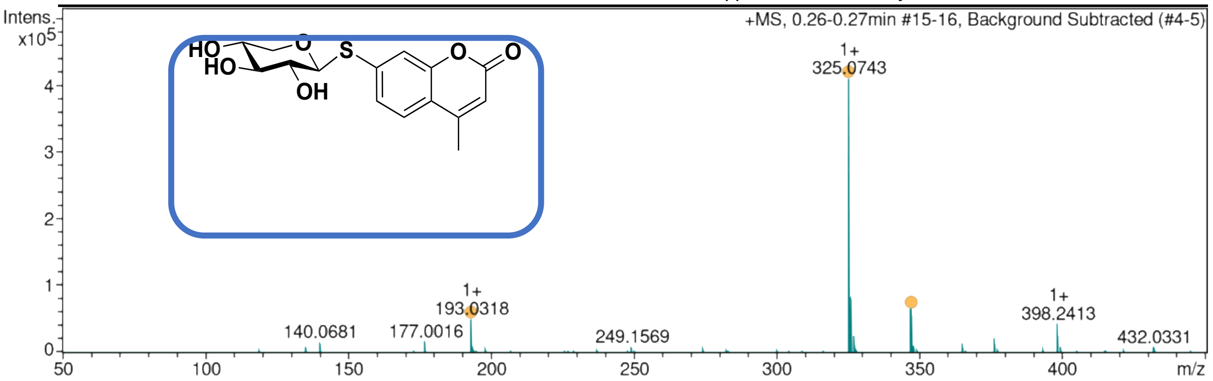

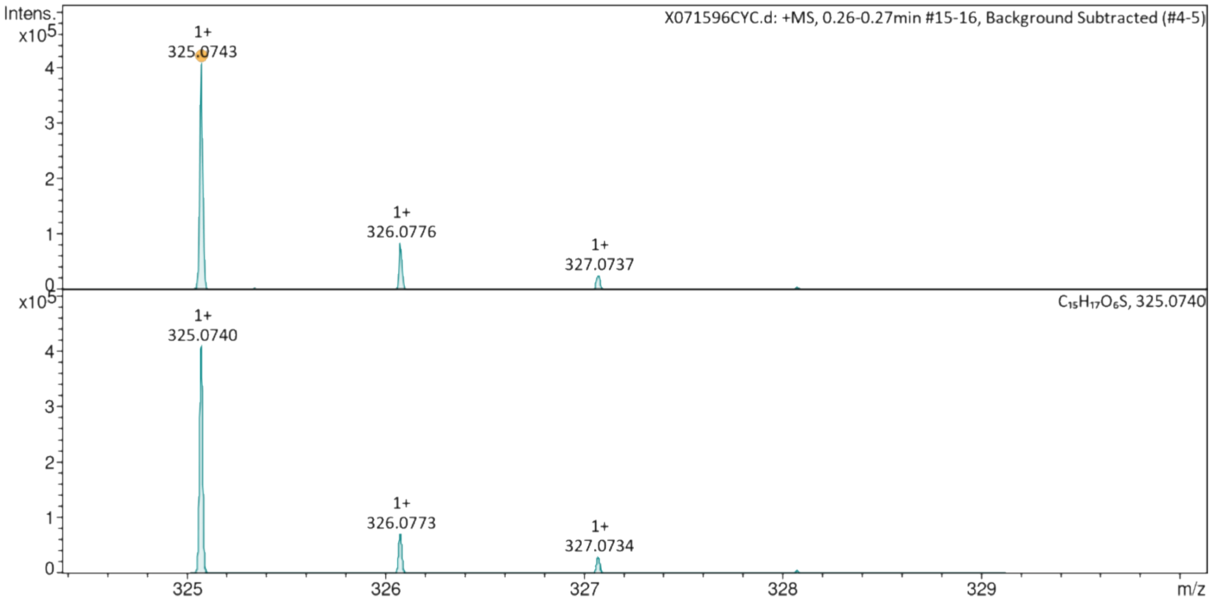

Meas. m/z	z	#	Ion Formula	m/z	err [ppm]	mSigma	rdb	e⁻ Conf
193.031796	1+	1	C10H9O2S	193.031777	-0.1	6.8	7.0	even
325.074328	1+	1	C15H17O6S	325.074036	-0.9	15.8	8.0	even
347.055977	1+	1	C15H16NaO6S	347.055980	0.0	6.4	8.0	even

Synthèse et Analyse pour La Santé, l'Agronomie et bien-être
Spectrométrie de Masse Haute Résolution

**Analysis Info**		Acquisition Date	01/03/2023 22:17:29
Sample Name	**Xyl-Coumarin**	Instrument / Ser#	maXis 255552.00086
Analysis Name	X071597CYC.d	Method	Negatif.m

Acquisition Parameter

Source Type	ESI	Ion Polarity	Negative	Set Nebulizer	0.6 Bar
Scan Begin	50 m/z	Set Capillary	4000 V	Set Dry Heater	200 °C
Scan End	3000 m/z	Set Collision Cell RF	500.0 Vpp	Set Dry Gas	7.0 l/min

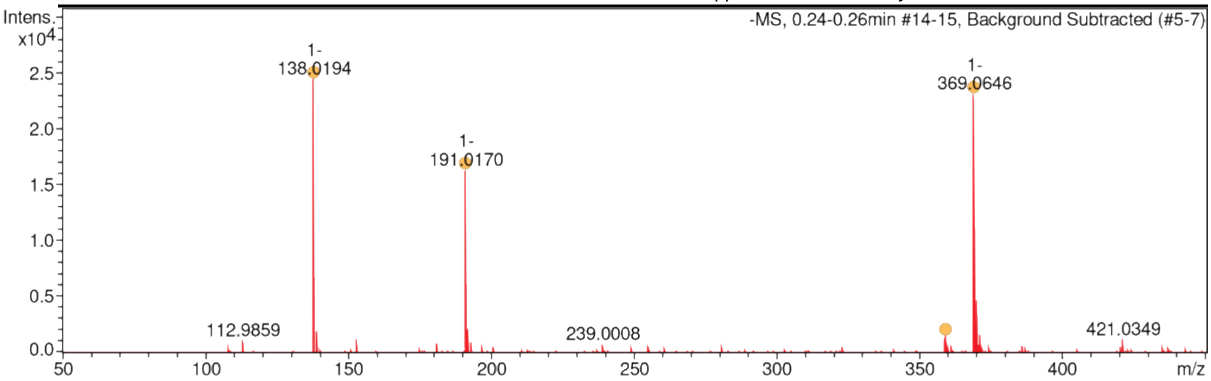

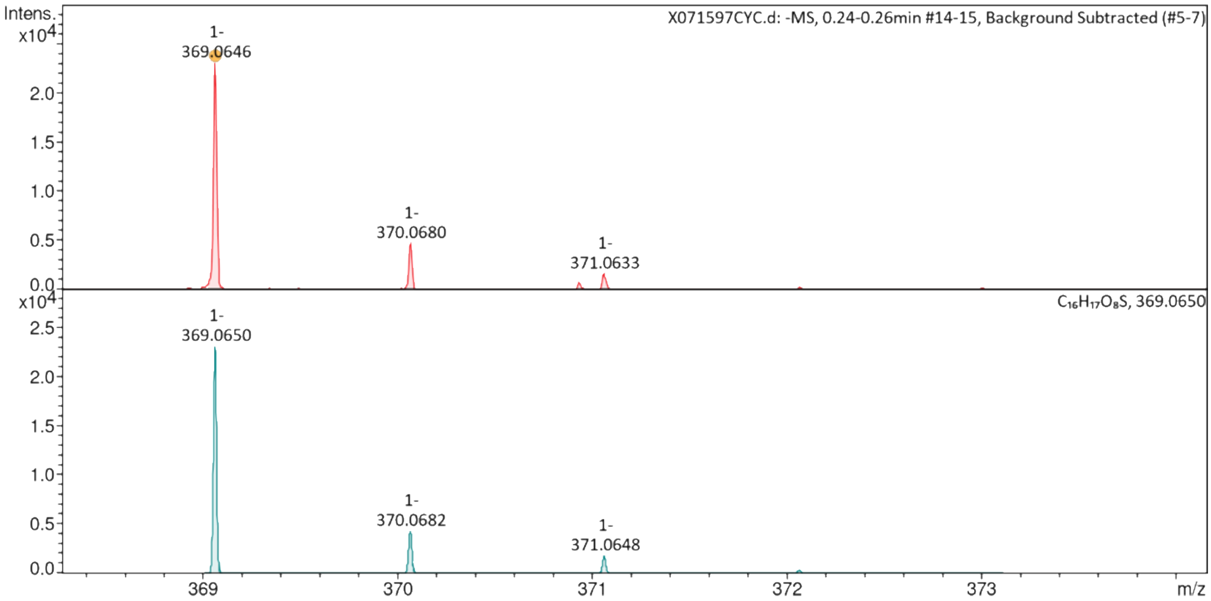

Meas. m/z	#	Ion Formula	m/z	err [ppm]	mSigma	rdb	e⁻ Conf	N-Rule
138.019403	1	C6H4NO3	138.019667	1.9	6.9	5.0	even	ok
191.016976	1	C10H7O2S	191.017224	1.3	6.6	7.0	even	ok
359.034861	1	C16H11N2O6S	359.034331	-1.5	213.1	12.0	even	ok
369.064579	1	C16H17O8S	369.064962	1.0	11.3	8.0	even	ok

Figure S20: MS spectra (positive and negative mode) of product **S-2**

SALSA

Synthèse et Analyse pour La Santé, l'Agronomie et le Bien-être
Spectrométrie de Masse Haute Résolution

IBiSA Infrastructures en Biologie Santé et Agronomie

**Analysis Info**		Acquisition Date	08/03/2023 11:37:47
Sample Name	**Glc-Coumarin**	Instrument / Ser#	maXis 255552.00086
Analysis Name	X071636CYC.d	Method	positif-6.m

Acquisition Parameter

Source Type	ESI	Ion Polarity	Positive	Set Nebulizer	0.6 Bar
Scan Begin	50 m/z	Set Capillary	4500 V	Set Dry Heater	200 °C
Scan End	2500 m/z	Set Collision Cell RF	1800.0 Vpp	Set Dry Gas	7.0 l/min

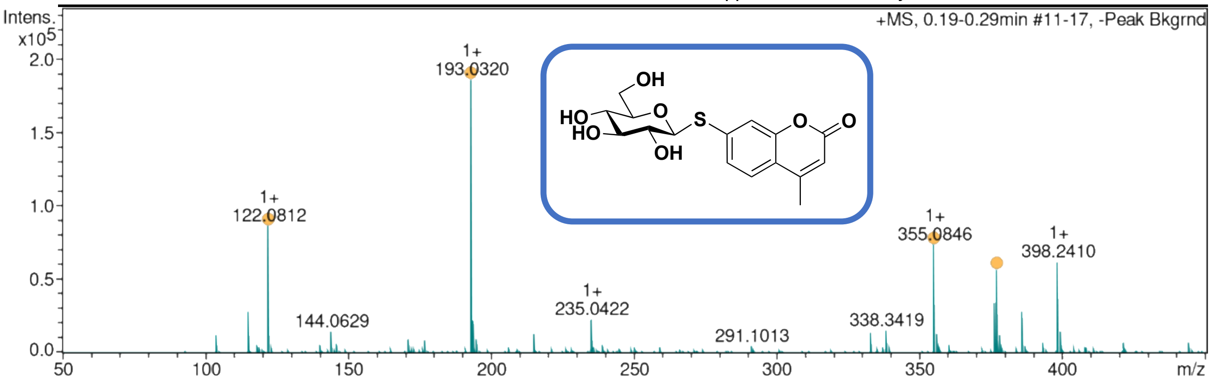

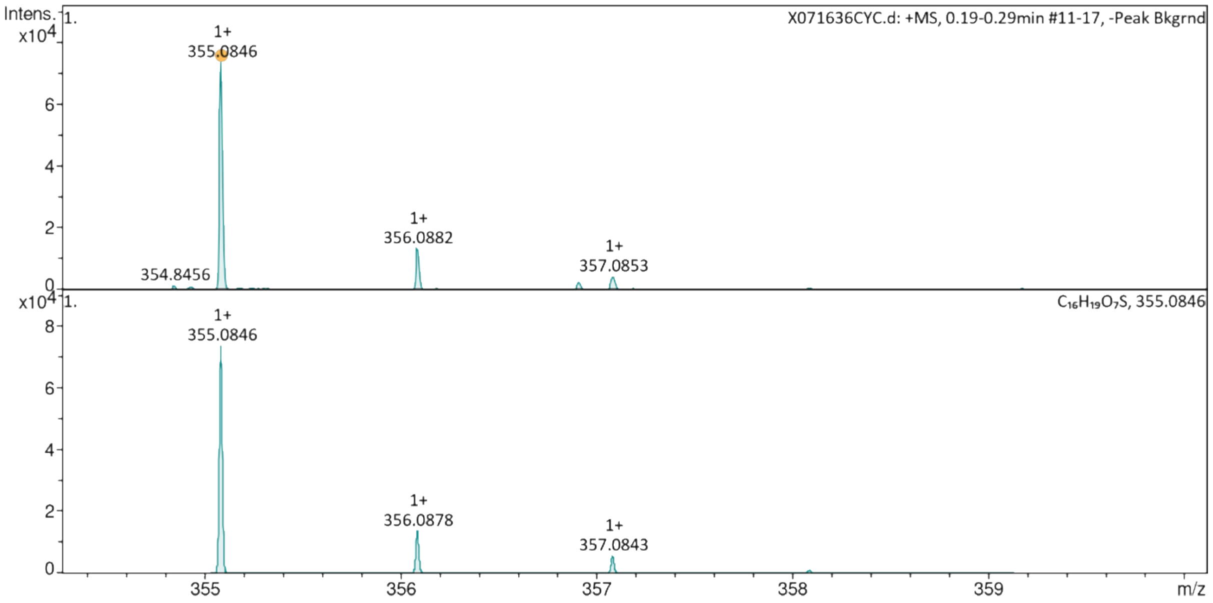

Meas. m/z	z	#	Ion Formula	m/z	err [ppm]	mSigma	rdb	e⁻ Conf
122.081157	1+	1	$C_4H_{12}NO_3$	122.081170	0.1	6.4	0.0	even
193.031977	1+	1	$C_{10}H_9O_2S$	193.031777	-1.0	1.6	7.0	even
355.084597	1+	1	$C_{16}H_{19}O_7S$	355.084600	0.0	10.9	8.0	even
377.066610	1+	1	$C_{16}H_{18}NaO_7S$	377.066545	-0.2	18.3	8.0	even

**Analysis Info**		Acquisition Date	08/03/2023 13:52:59
Sample Name	**Glc-Coumarin**	Instrument / Ser#	maXis 255552.00086
Analysis Name	X071637CYC_17867.d	Method	Negatif.m

**Acquisition Parameter**					
Source Type	ESI	Ion Polarity	Negative	Set Nebulizer	0.6 Bar
Scan Begin	50 m/z	Set Capillary	4000 V	Set Dry Heater	200 °C
Scan End	3000 m/z	Set Collision Cell RF	500.0 Vpp	Set Dry Gas	7.0 l/min

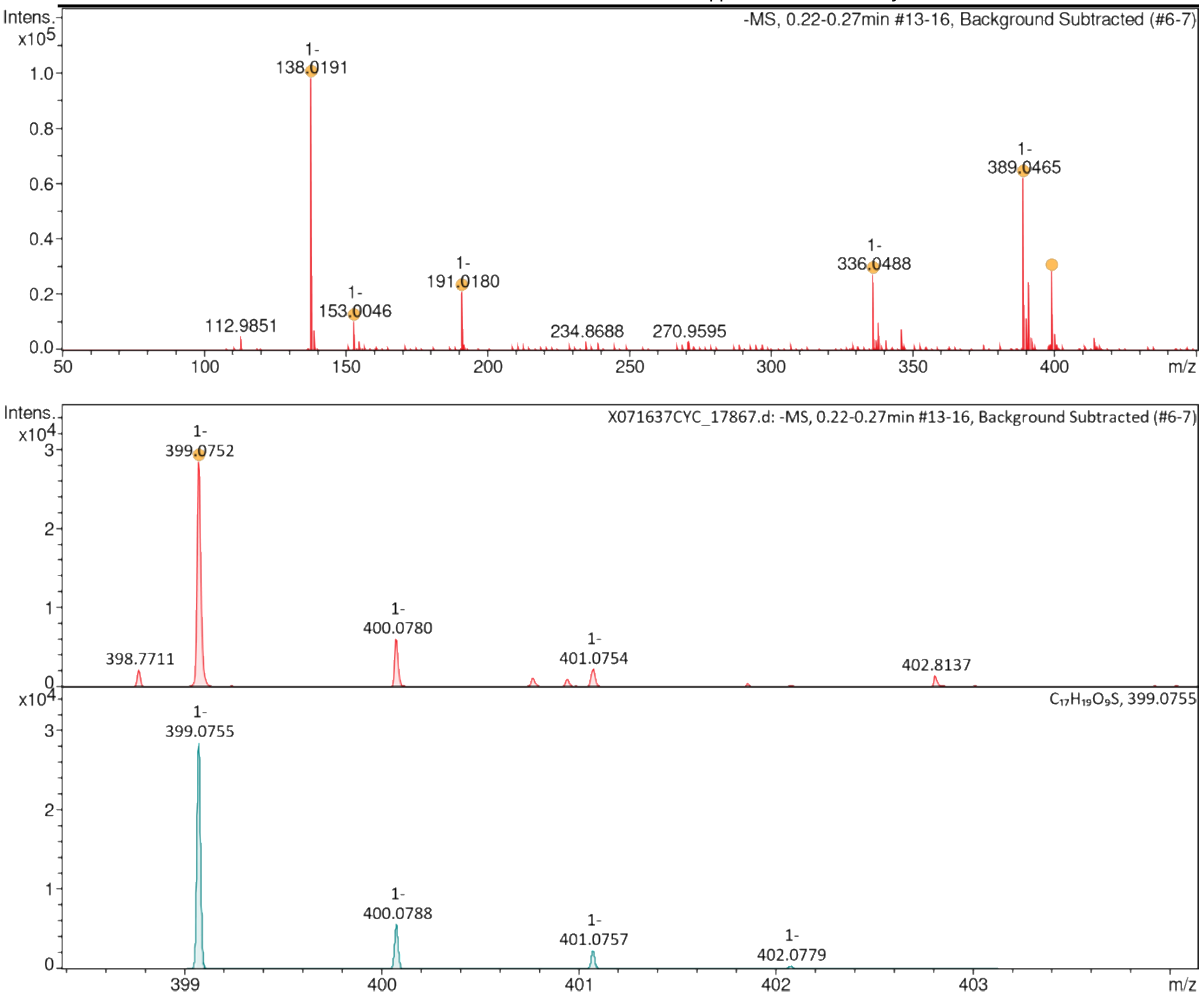

Meas. m/z	#	Ion Formula	m/z	err [ppm]	mSigma	rdb	e¯ Conf	N-Rule
138.019086	1	C6H4NO3	138.019667	4.2	3.2	5.0	even	ok
153.004579	1	C3H5O7	153.004076	-3.3	48.0	1.0	even	ok
191.018004	1	C10H7O2S	191.017224	-4.1	16.8	7.0	even	ok
336.048765	1	C12H15ClNO8	336.049168	1.2	9.0	5.0	even	ok
389.046463	1	C16H18ClO7S	389.046725	0.7	2.4	7.0	even	ok
399.075231	1	C17H19O9S	399.075527	0.7	9.9	8.0	even	ok

Figure S21: MS spectra (positive and negative mode) of product **S-3**

SALSA Synthèse et Analyse pour La Santé, l'Agronomie et le bien-être
Spectrométrie de Masse Haute Résolution

IBiSA Infrastructures en Biologie Santé et Agronomie

Analysis Info

Sample Name	**Gal_Coumarin**	Acquisition Date	20/02/2024 17:29:43
Analysis Name	X076634CYC.d	Instrument / Ser#	maXis 255552.00086
		Method	negatif-dgp-l+sl.m

Acquisition Parameter

Source Type	ESI	Ion Polarity	Negative	Set Nebulizer	0.6 Bar
Scan Begin	50 m/z	Set Capillary	4000 V	Set Dry Heater	200 °C
Scan End	3000 m/z	Set Collision Cell RF	500.0 Vpp	Set Dry Gas	7.0 l/min

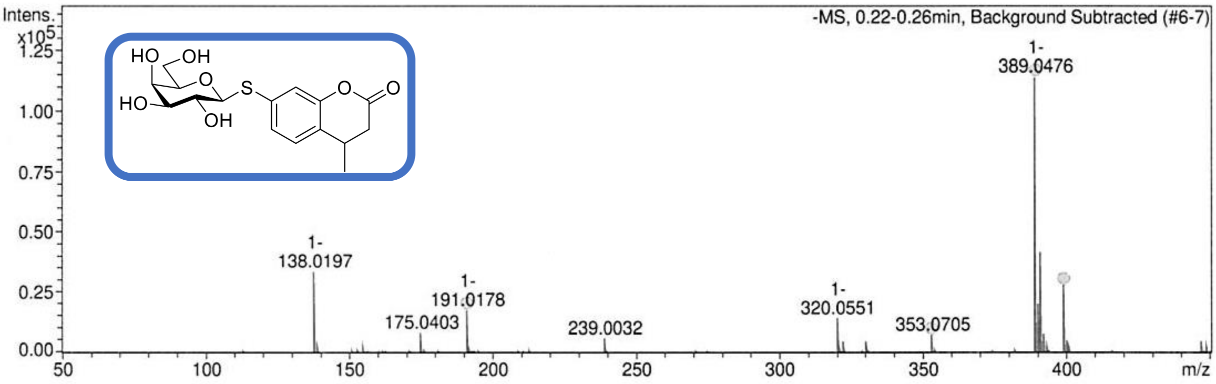

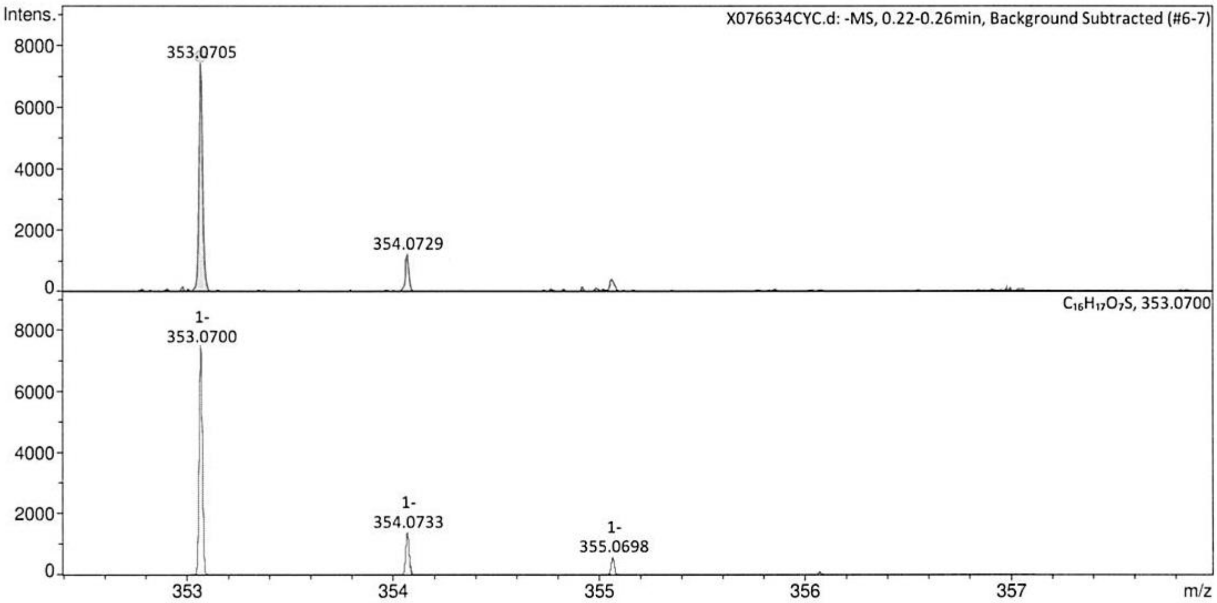

Meas. m/z	#	Ion Formula	m/z	err [ppm]	mSigma	rdb	e⁻ Conf	N-Rule
191.017826	1	C10H7O2S	191.017224	-3.2	13.2	7.0	even	ok
353.070454	1	C16H17O7S	353.070047	-1.2	39.6	8.0	even	ok
389.047626	1	C16H18ClO7S	389.046725	-2.3	12.5	7.0	even	ok
399.076168	1	C17H19O9S	399.075527	-1.6	12.4	8.0	even	ok

icoa
Institut de Chimie Organique et Analytique
UMR 7311 - Université d'Orléans - CNRS
BP 6759 - rue de Chartres
F-45067 ORLEANS Cedex 2
cyril.colas@univ-orleans.fr
+33 (0)2 38 49 46 61

printed: 21/02/2024 19:35:31

Figure S22: MS spectra (negative mode) of product **S-4**

Analysis Info

Sample Name	**Fuc-Coumarin**	Acquisition Date	08/03/2023 12:35:25
Analysis Name	X071634CYC_17862.d	Instrument / Ser#	maXis 255552.00086
		Method	positif-6.m

Acquisition Parameter

Source Type	ESI	Ion Polarity	Positive	Set Nebulizer	0.6 Bar
Scan Begin	50 m/z	Set Capillary	4500 V	Set Dry Heater	200 °C
Scan End	2500 m/z	Set Collision Cell RF	1800.0 Vpp	Set Dry Gas	7.0 l/min

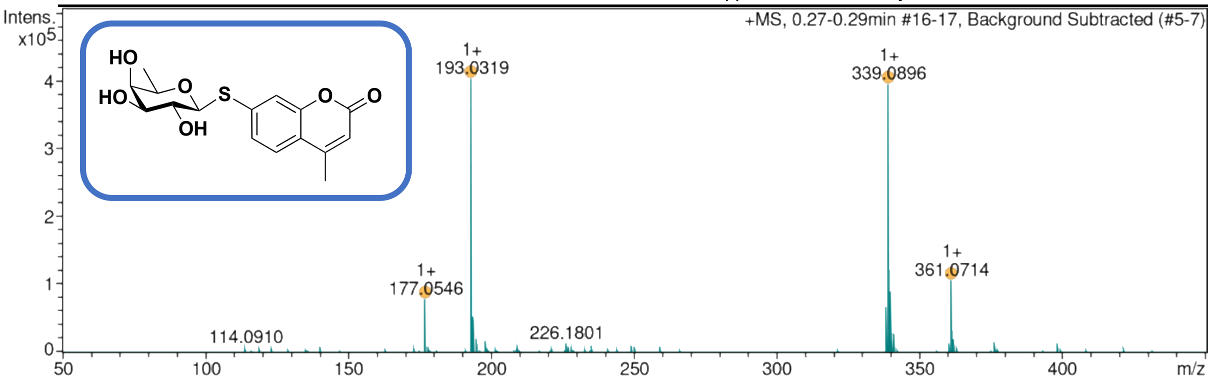

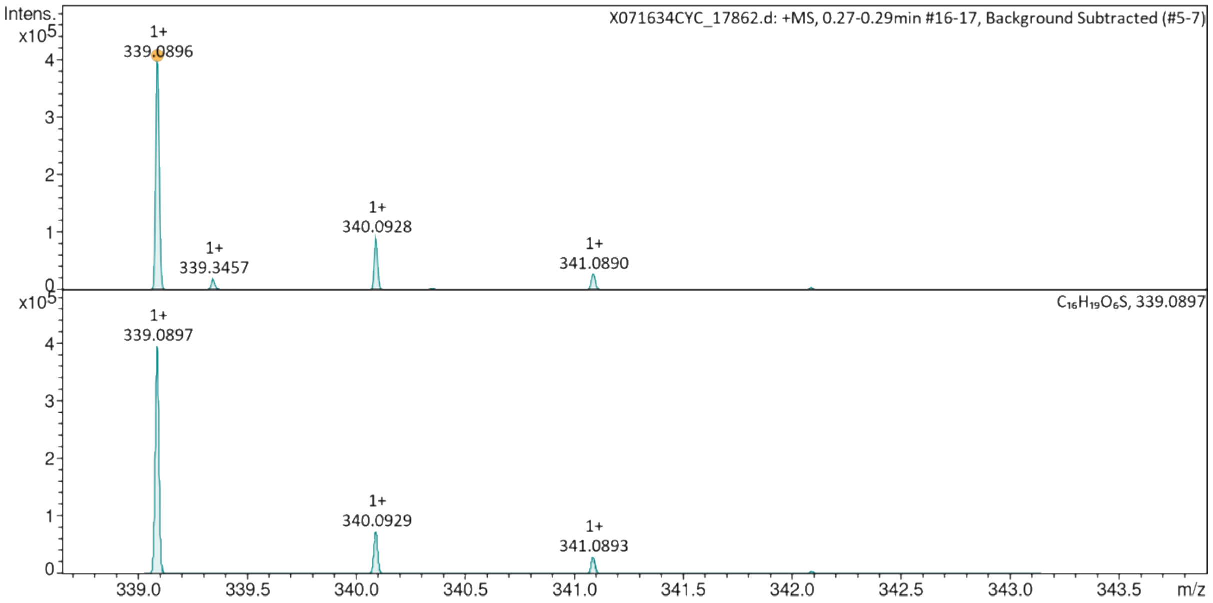

Meas. m/z	z	#	Ion Formula	m/z	err [ppm]	mSigma	rdb	e¯ Conf
177.054598	1+	1	C10H9O3	177.054621	0.1	3.7	7.0	even
193.031913	1+	1	C10H9O2S	193.031777	-0.7	7.5	7.0	even
339.089599	1+	1	C16H19O6S	339.089686	0.3	21.5	8.0	even
361.071356	1+	1	C16H18NaO6S	361.071630	0.8	10.2	8.0	even

Analysis Info

Sample Name	**Fuc-Coumarin**	Acquisition Date	08/03/2023 13:45:02
		Instrument / Ser#	maXis 255552.00086
Analysis Name	X071635CYC.d	Method	Negatif.m

Acquisition Parameter

Source Type	ESI	Ion Polarity	Negative	Set Nebulizer	0.6 Bar
Scan Begin	50 m/z	Set Capillary	4000 V	Set Dry Heater	200 °C
Scan End	3000 m/z	Set Collision Cell RF	500.0 Vpp	Set Dry Gas	7.0 l/min

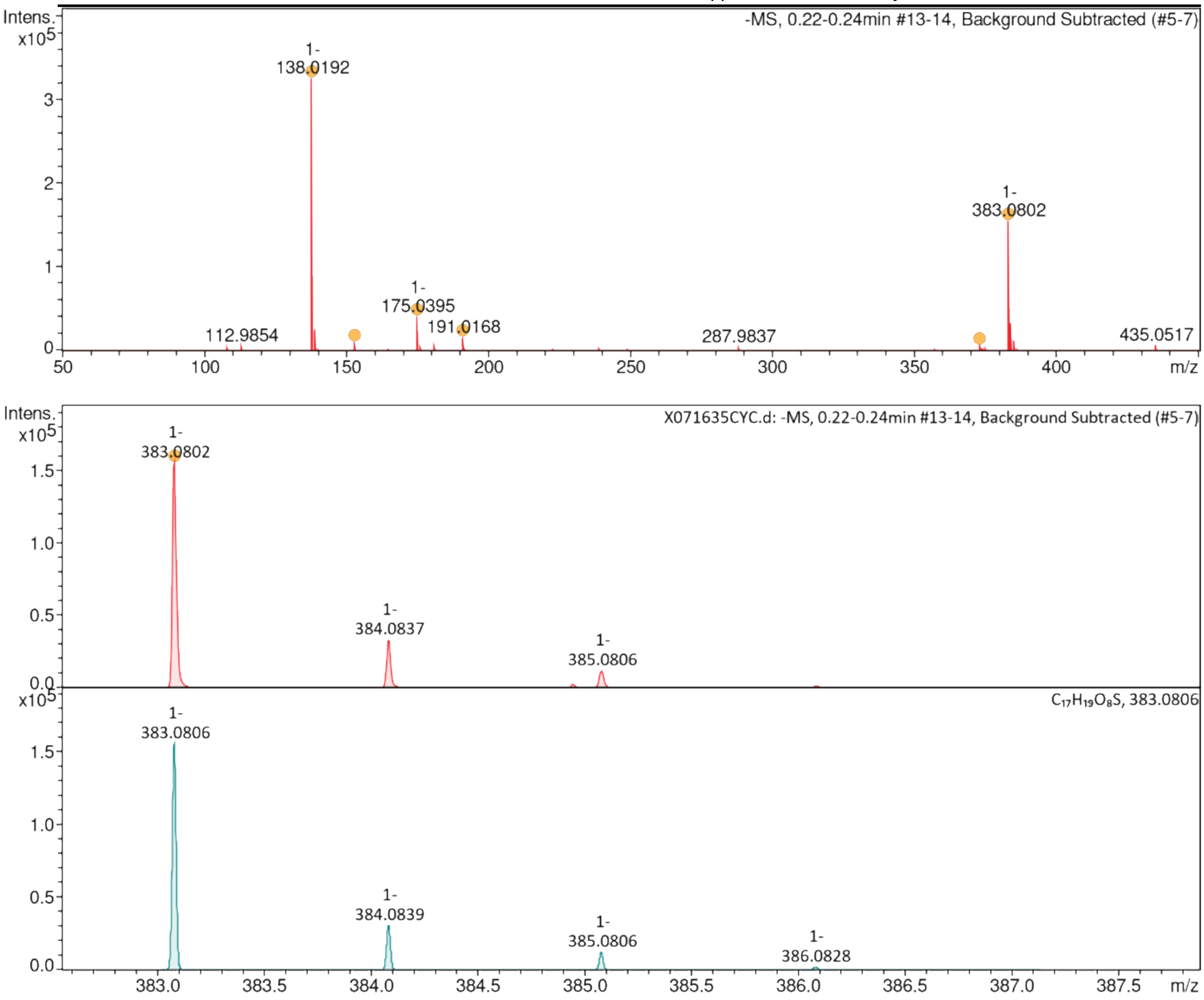

Meas. m/z	#	Ion Formula	m/z	err [ppm]	mSigma	rdb	e⁻ Conf	N-Rule
138.019167	1	C6H4NO3	138.019667	3.6	5.2	5.0	even	ok
153.004429	1	C3H5O7	153.004076	-2.3	43.1	1.0	even	ok
175.039515	1	C10H7O3	175.040068	3.2	6.3	7.0	even	ok
191.016828	1	C10H7O2S	191.017224	2.1	5.6	7.0	even	ok
373.052003	1	C16H18ClO6S	373.051811	-0.5	48.9	7.0	even	ok
383.080191	1	C17H19O8S	383.080612	1.1	8.3	8.0	even	ok

Figure S23: MS spectra (positive and negative mode) of product **S-5**

Synthèse et Analyse pour La Santé, l'Agronomie btdn-être
Spectrométrie de Masse Haute Résolution

**Analysis Info**		Acquisition Date	26/04/2023 13:44:50
Sample Name	**Gal-Coumarin**	Instrument / Ser#	maXis 255552.00086
Analysis Name	X072559CYC.d	Method	positif-6.m

Acquisition Parameter

Source Type	ESI	Ion Polarity	Positive	Set Nebulizer	0.6 Bar
Scan Begin	50 m/z	Set Capillary	4500 V	Set Dry Heater	200 °C
Scan End	2500 m/z	Set Collision Cell RF	1800.0 Vpp	Set Dry Gas	7.0 l/min

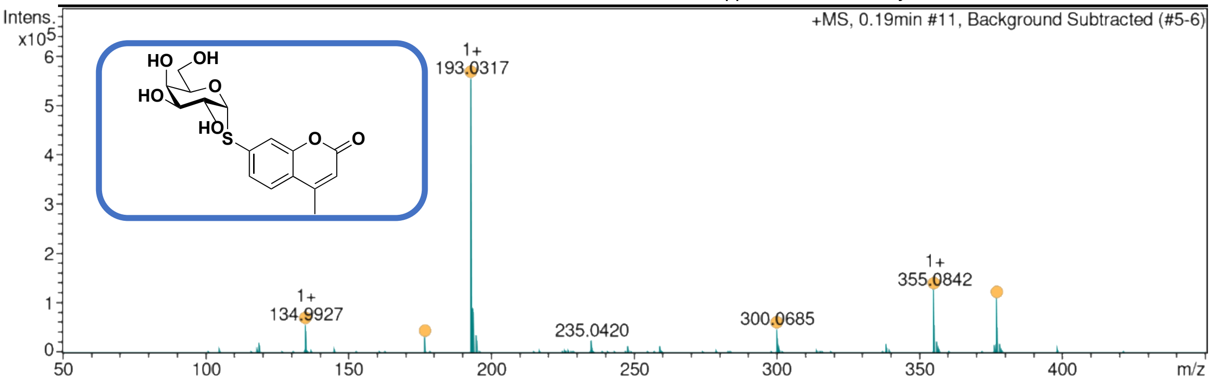

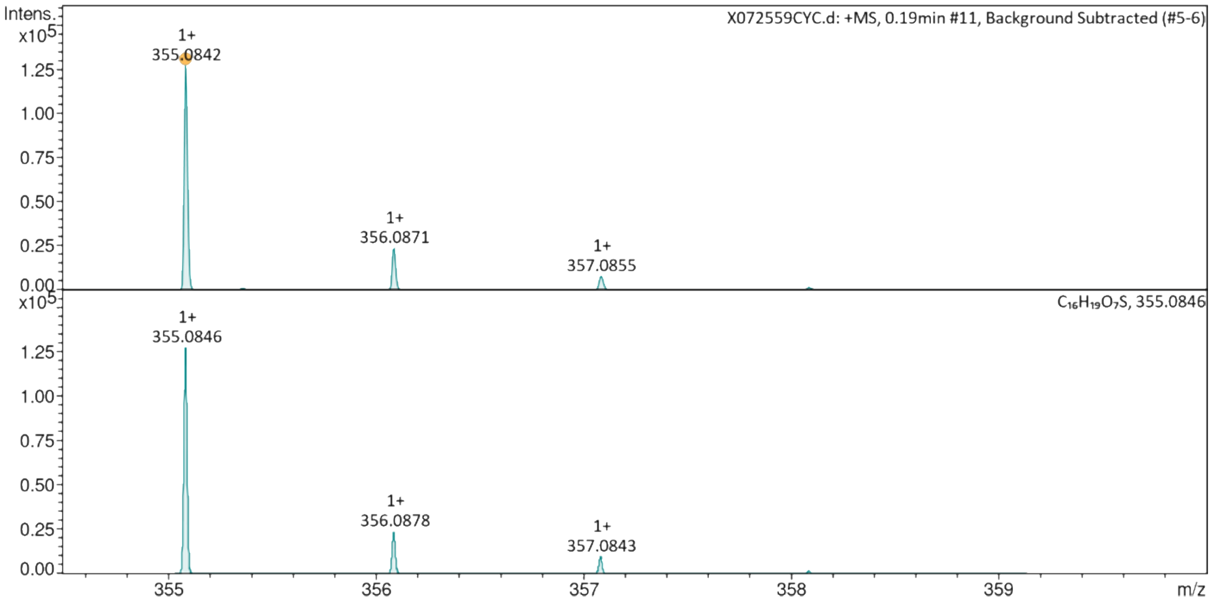

Meas. m/z	z	#	Ion Formula	m/z	err [ppm]	mSigma	rdb	e⁻ Conf
134.992651	1+	1	C3H3O6	134.992414	-1.8	57.8	3.0	even
177.001193	1+	1	C9H5O2S	177.000477	-4.0	46.8	8.0	even
193.031719	1+	1	C10H9O2S	193.031777	0.3	22.5	7.0	even
300.068473	1+	1	C16H14NO3S	300.068891	1.4	6.4	11.0	even
355.084229	1+	1	C16H19O7S	355.084600	1.0	7.1	8.0	even
377.066047	1+	1	C16H18NaO7S	377.066545	1.3	14.5	8.0	even

**Analysis Info**		Acquisition Date	26/04/2023 19:37:41
Sample Name	**Gal-Coumarin**	Instrument / Ser#	maXis 255552.00086
Analysis Name	X072560CYC.d	Method	Negatif.m

Acquisition Parameter

Source Type	ESI	Ion Polarity	Negative	Set Nebulizer	0.6 Bar
Scan Begin	50 m/z	Set Capillary	4000 V	Set Dry Heater	200 °C
Scan End	3000 m/z	Set Collision Cell RF	500.0 Vpp	Set Dry Gas	7.0 l/min

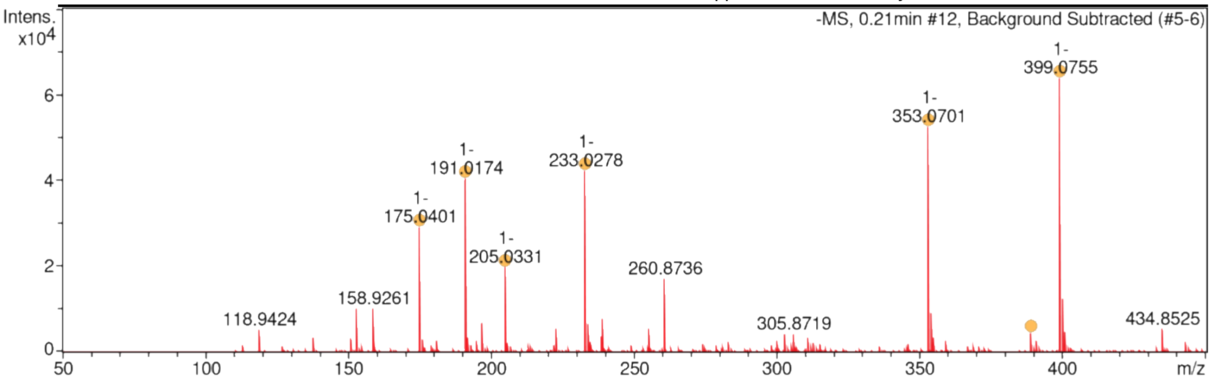

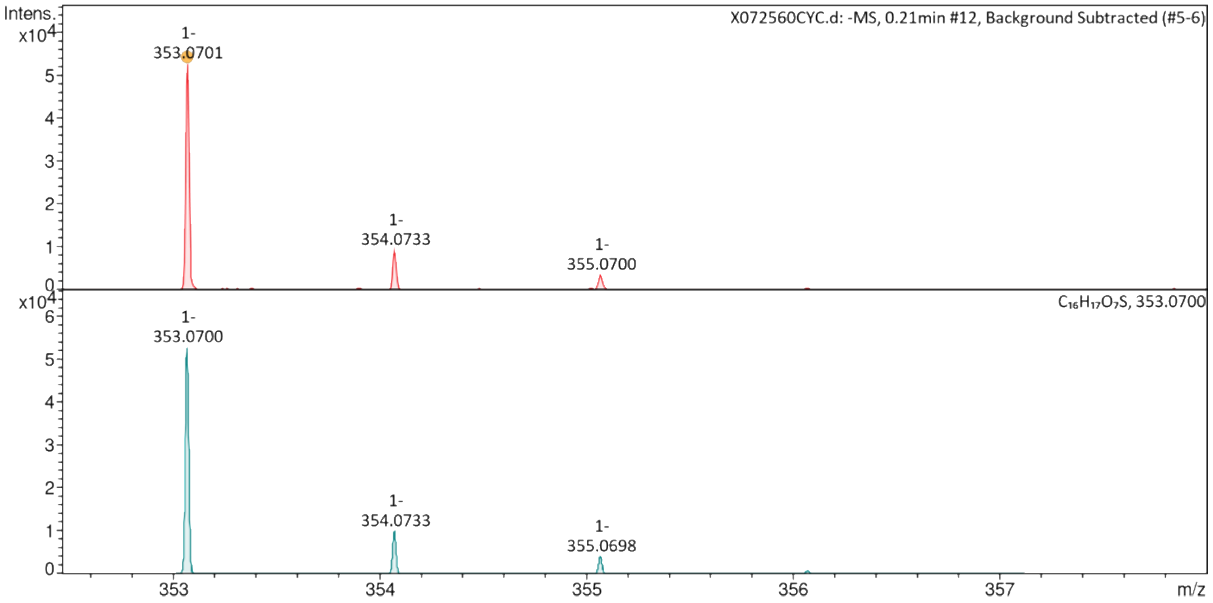

Meas. m/z	#	Ion Formula	m/z	err [ppm]	mSigma	rdb	e¯ Conf	N-Rule
175.040089	1	C10H7O3	175.040068	-0.1	9.2	7.0	even	ok
191.017446	1	C10H7O2S	191.017224	-1.2	18.0	7.0	even	ok
205.033068	1	C11H9O2S	205.032874	-0.9	10.3	7.0	even	ok
233.027833	1	C12H9O3S	233.027789	-0.2	8.6	8.0	even	ok
353.070147	1	C16H17O7S	353.070047	-0.3	10.1	8.0	even	ok
389.045792	1	C16H18ClO7S	389.046725	2.4	129.6	7.0	even	ok
399.075520	1	C17H19O9S	399.075527	0.0	7.8	8.0	even	ok

Figure S24: MS spectra (positive and negative mode) of product **S-6**

^{1}H and ^{13}C-NMR of undescribed products

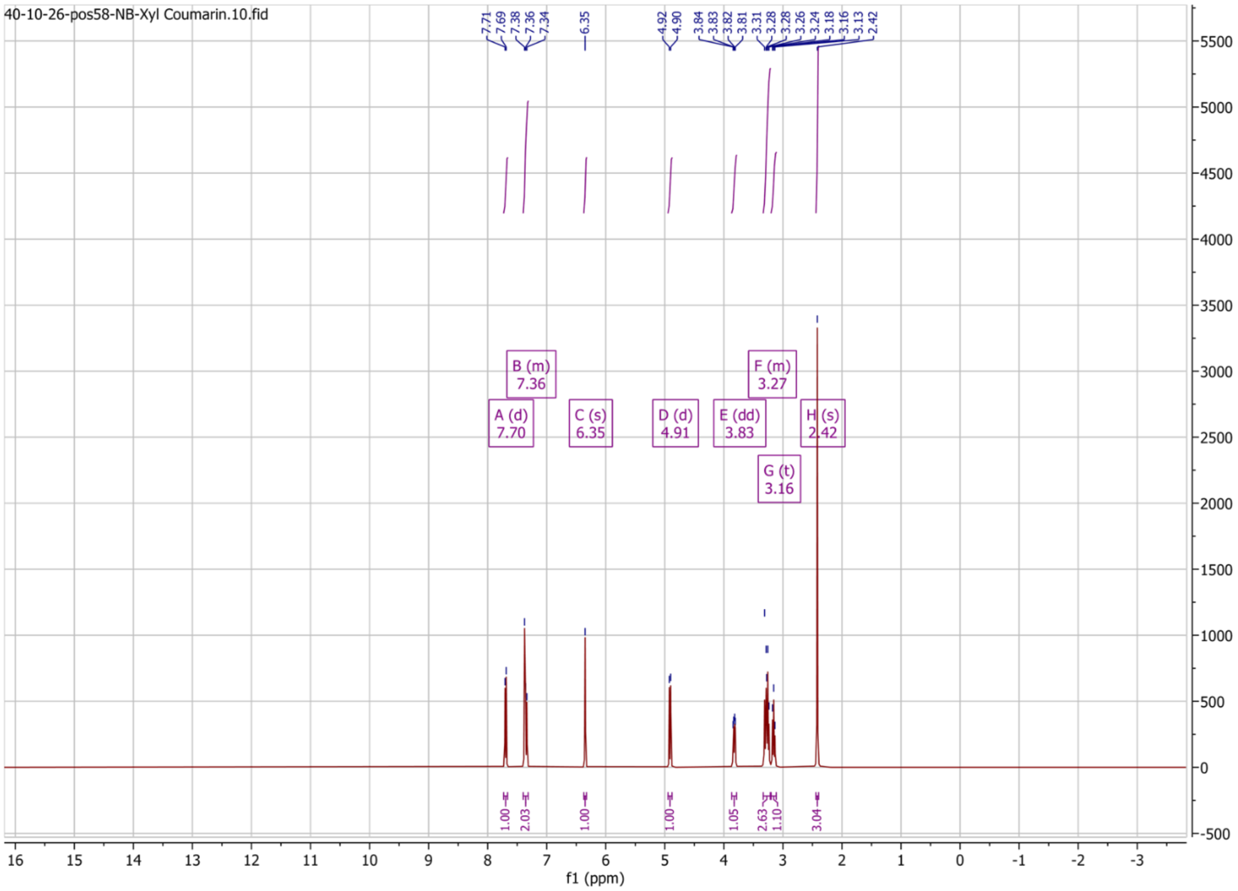

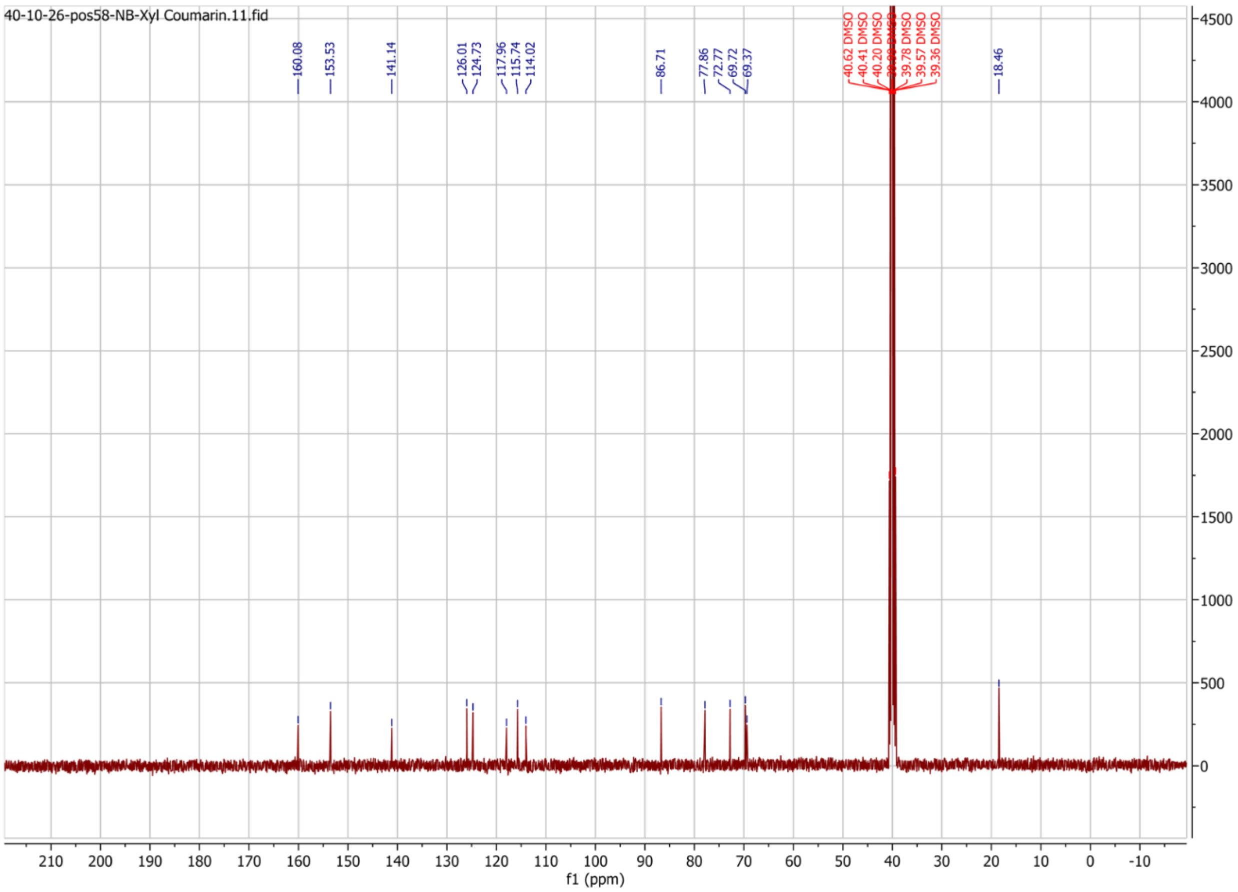

Figure S25. ^{1}H and ^{13}C-NMR of purified product **S-2**

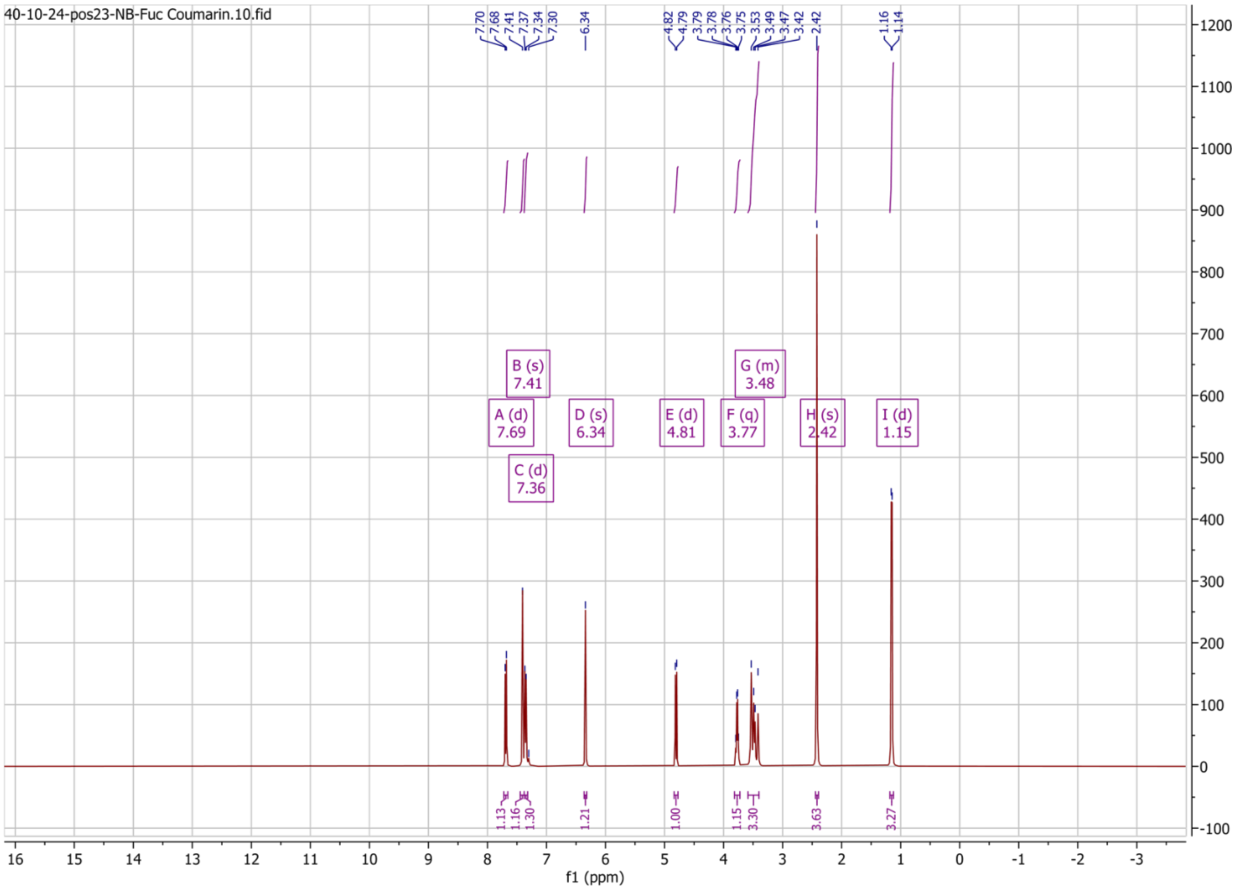

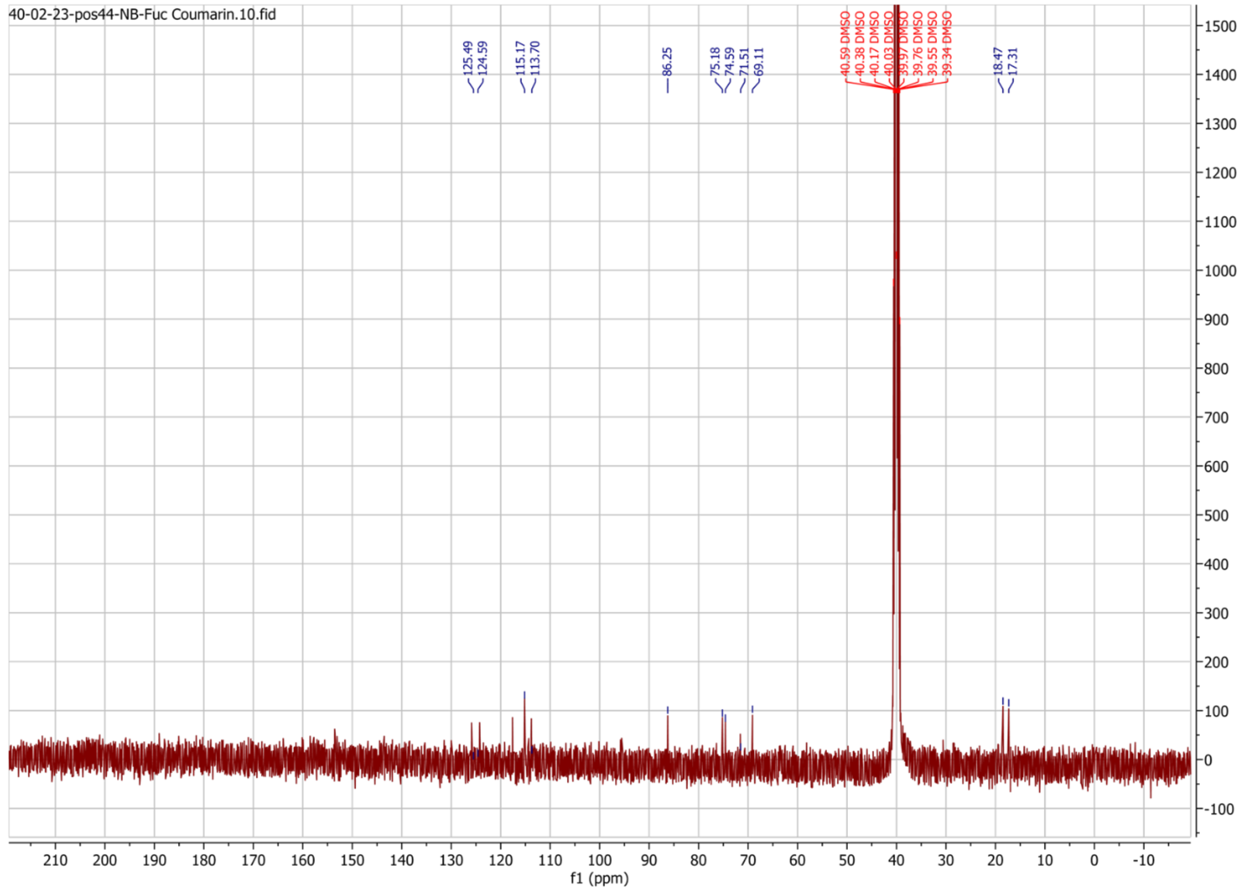

Figure S26. ^{1}H and ^{13}C-NMR of purified product **S-5**

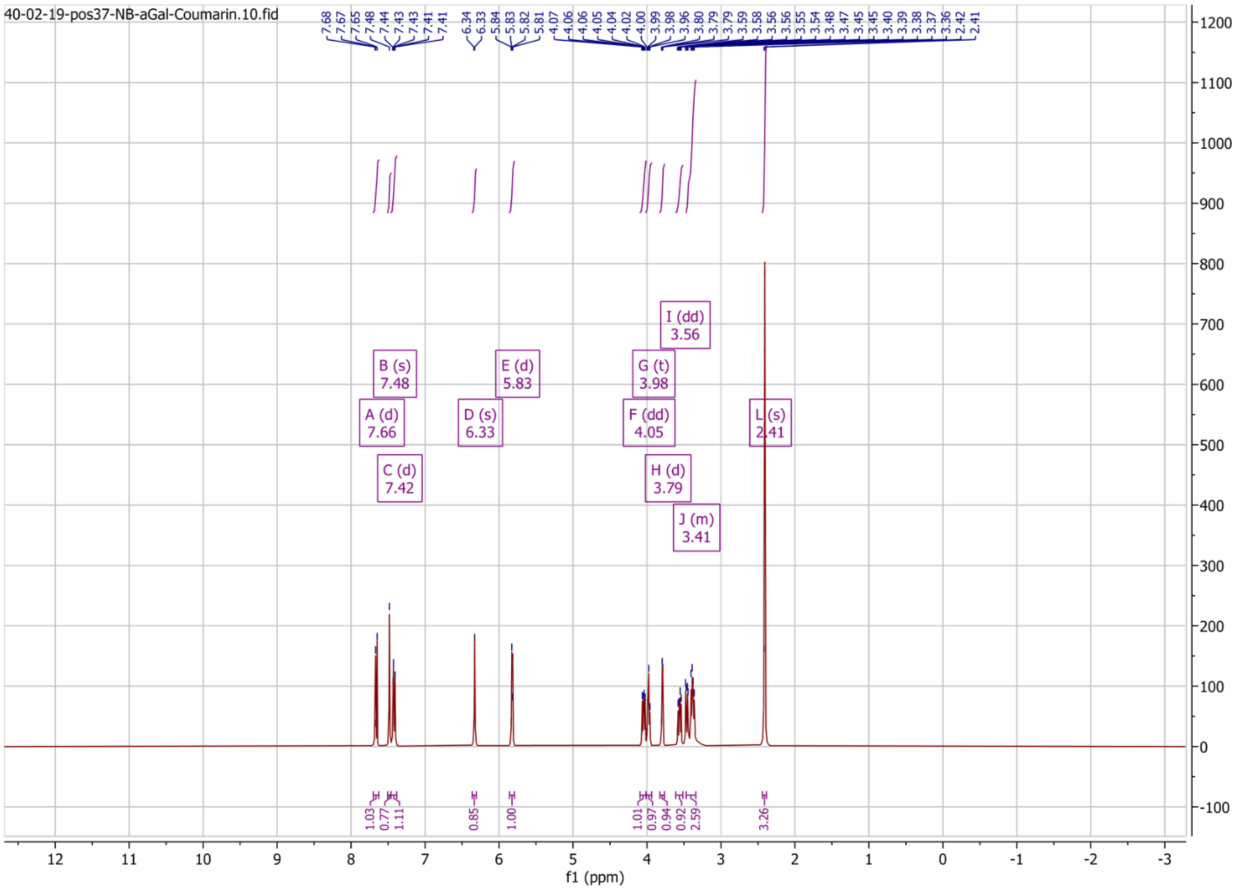

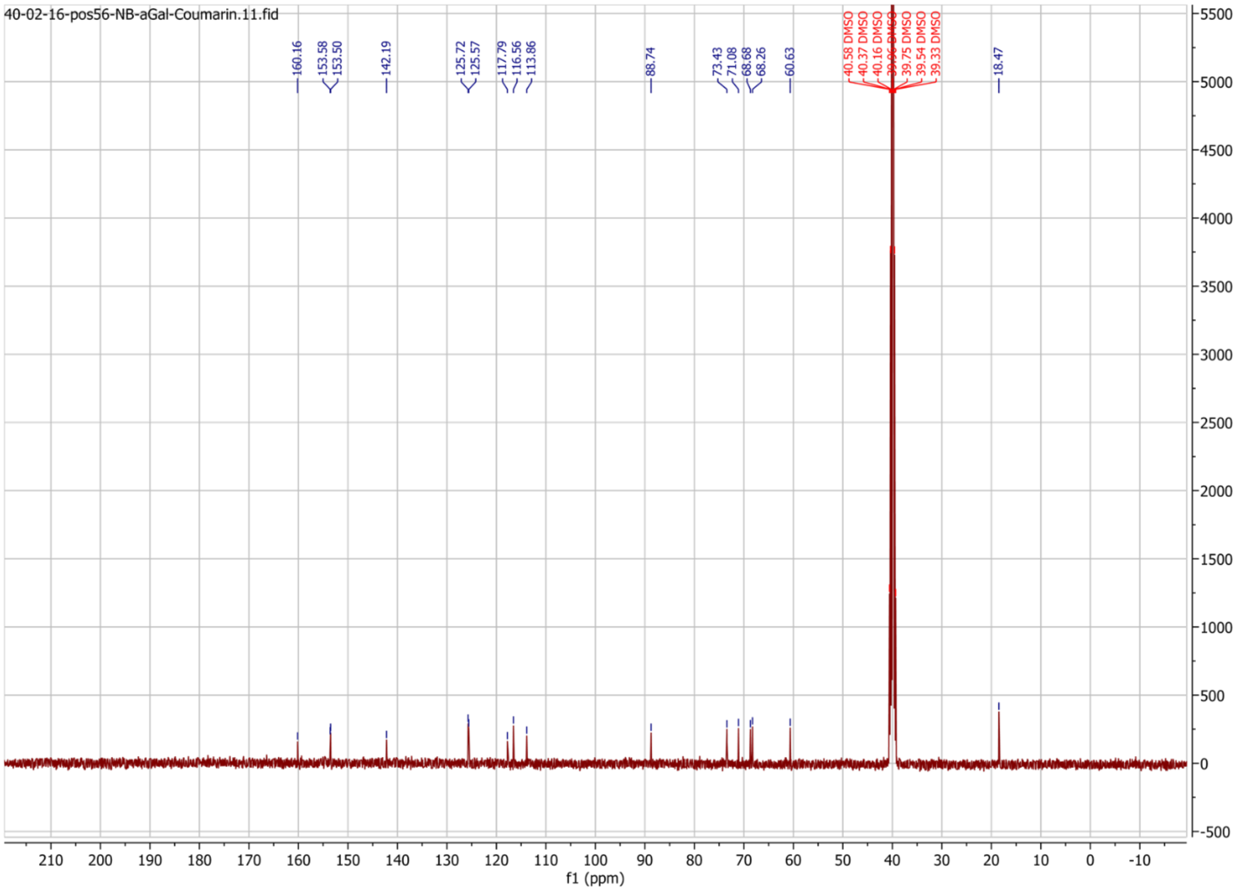

Figure S27. ^{1}H and ^{13}C-NMR of purified product **S-6**

Fluorescence analysis and spectra

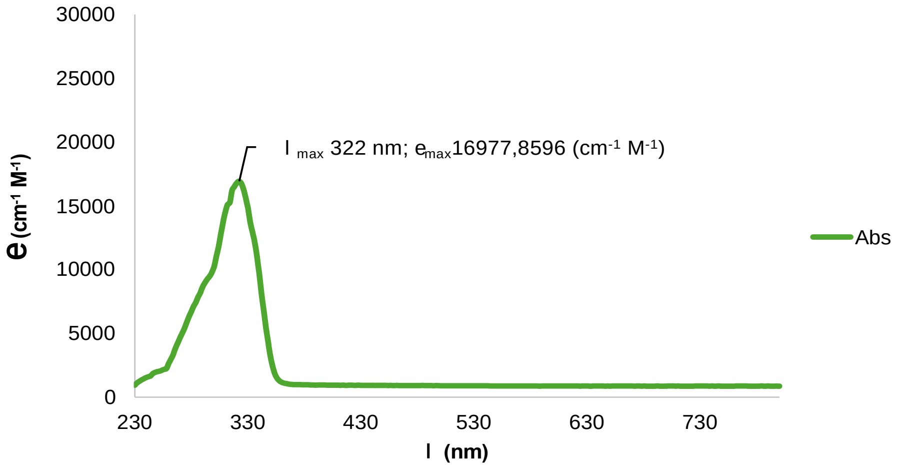

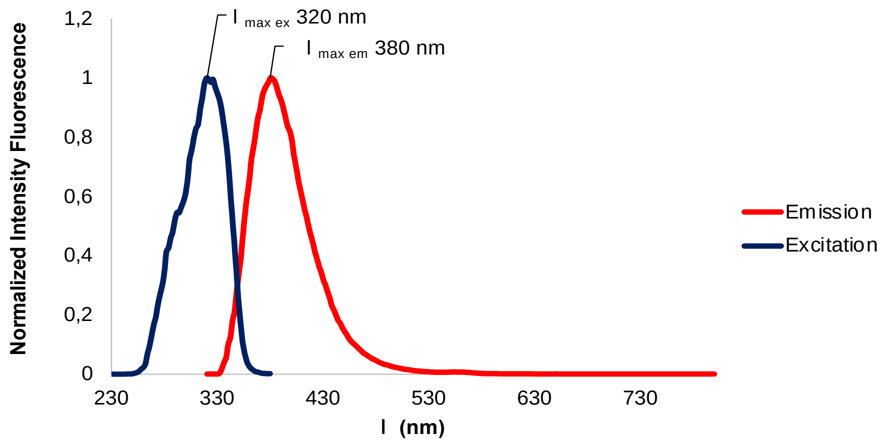

Figure S28. Absorbance and normalized fluorescence emission/excitation spectra of **4-MUB** 0.1 mM in DMF

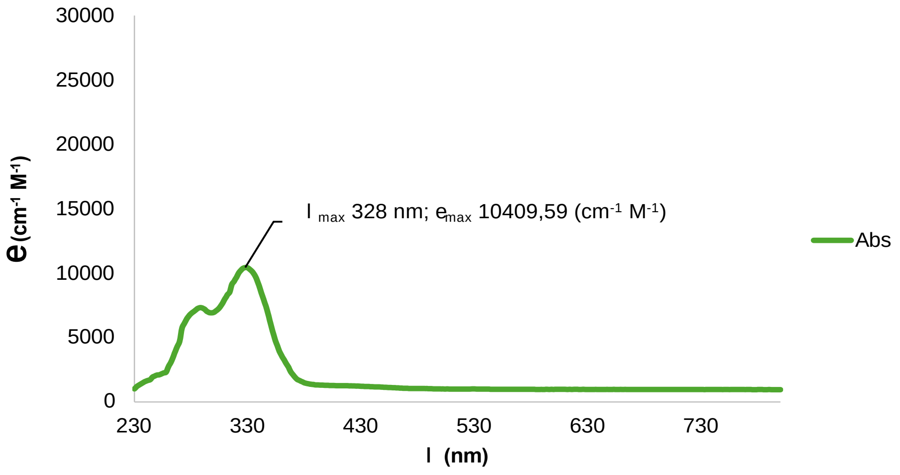

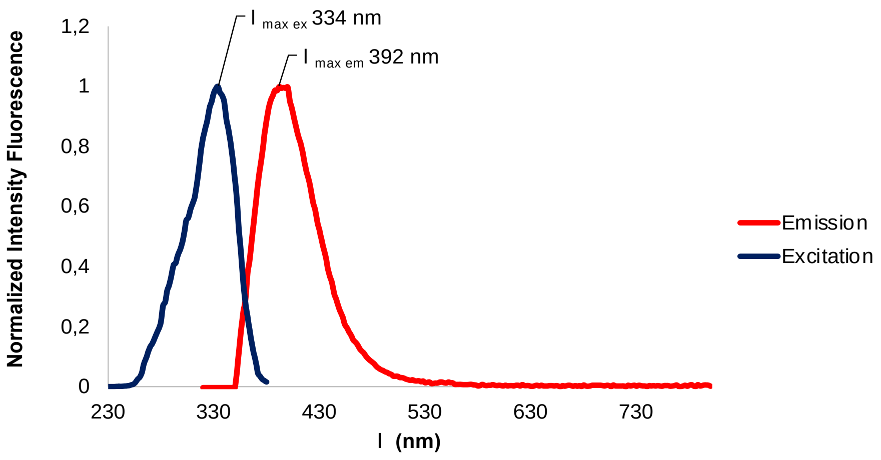

Figure S29. Absorbance and normalized fluorescence emission/excitation spectra of **7-MC** 0.1 mM + DTT 10 mM in DMF

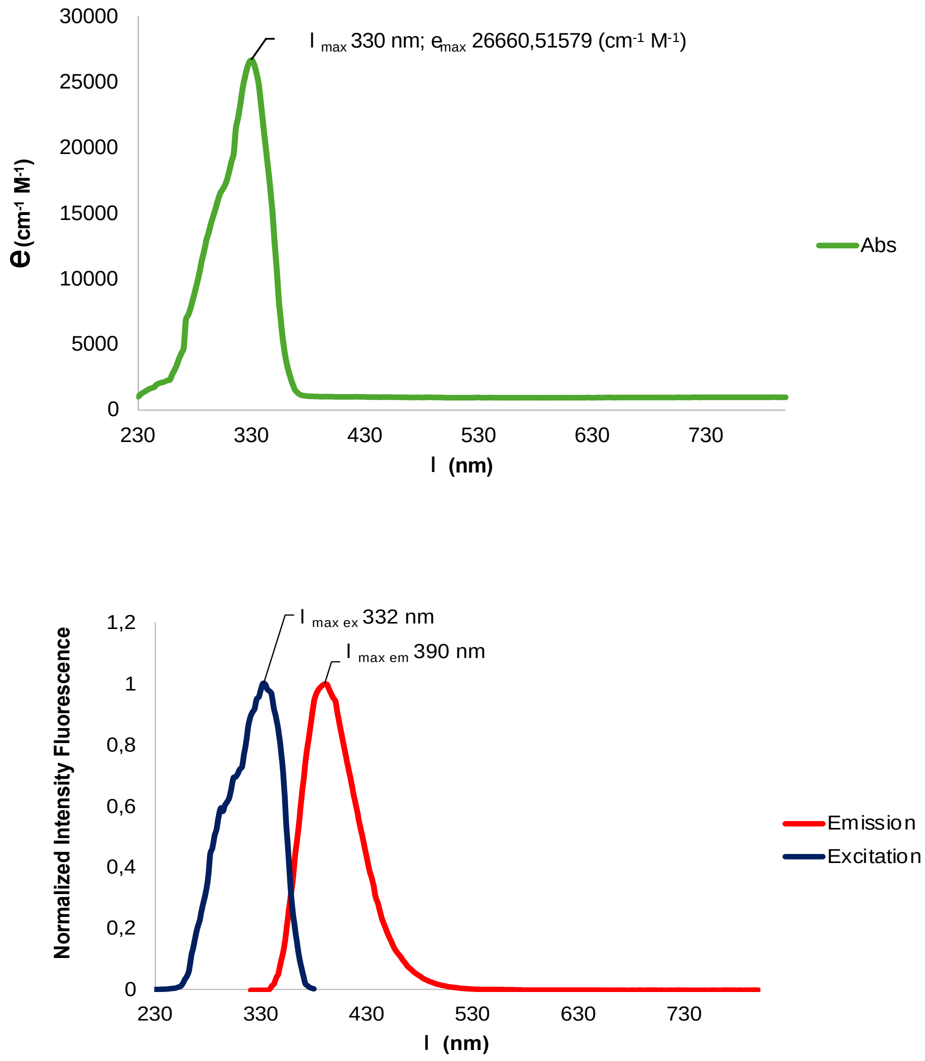

Figure S30. Absorbance and normalized fluorescence emission/excitation spectra of **S-1** 0.1 mM in DMF

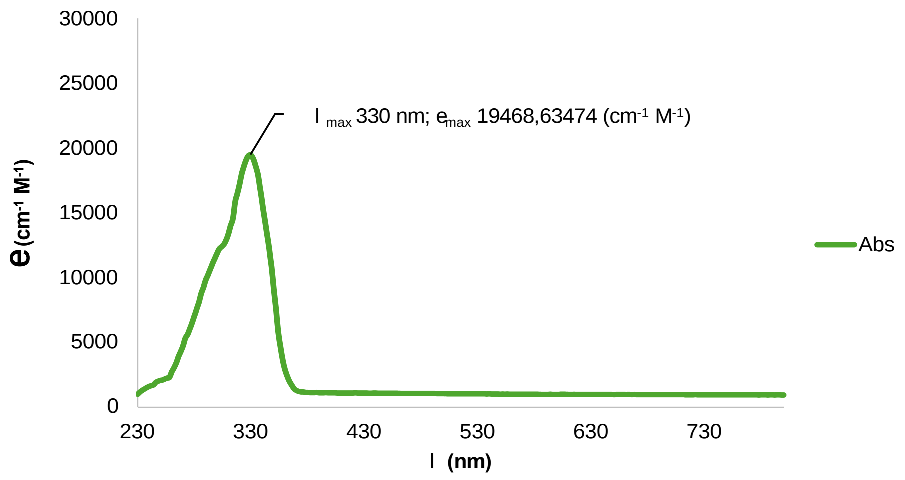

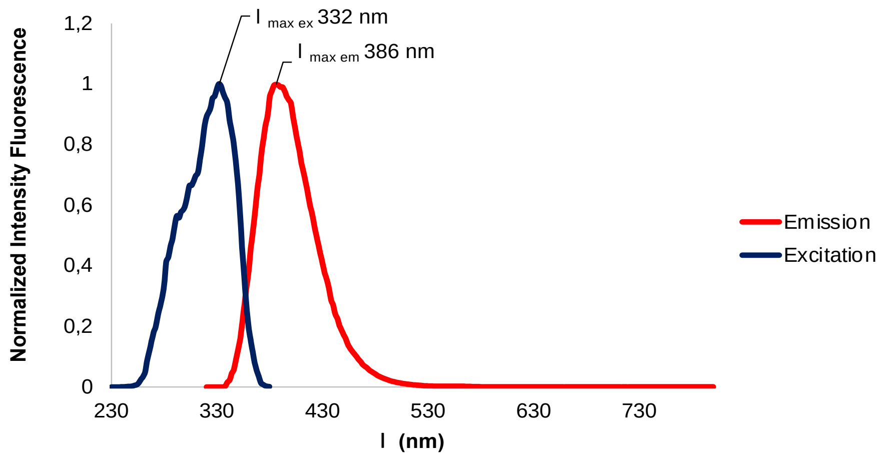

Figure S31. Absorbance and normalized fluorescence emission/excitation spectra of **S-2** 0.1mM in DMF

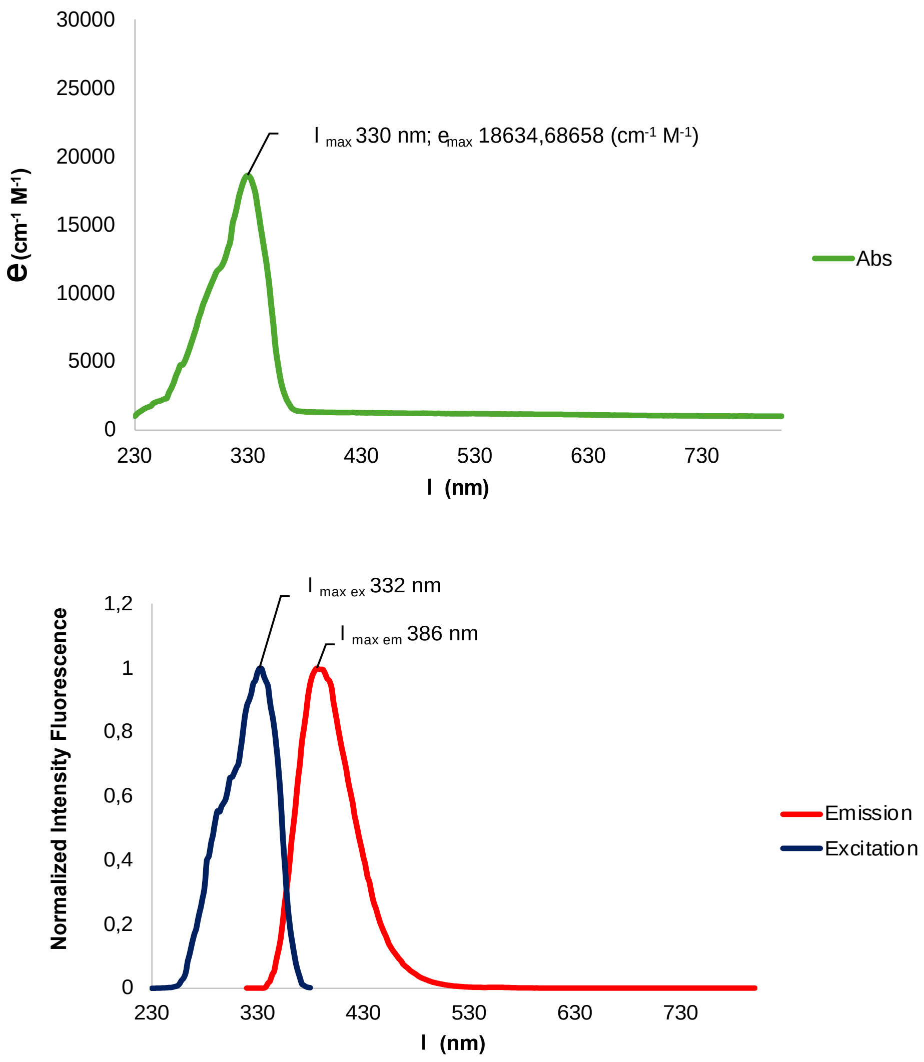

Figure S32. Absorbance and normalized fluorescence emission/excitation spectra of **S-3** 0.1 mM in DMF

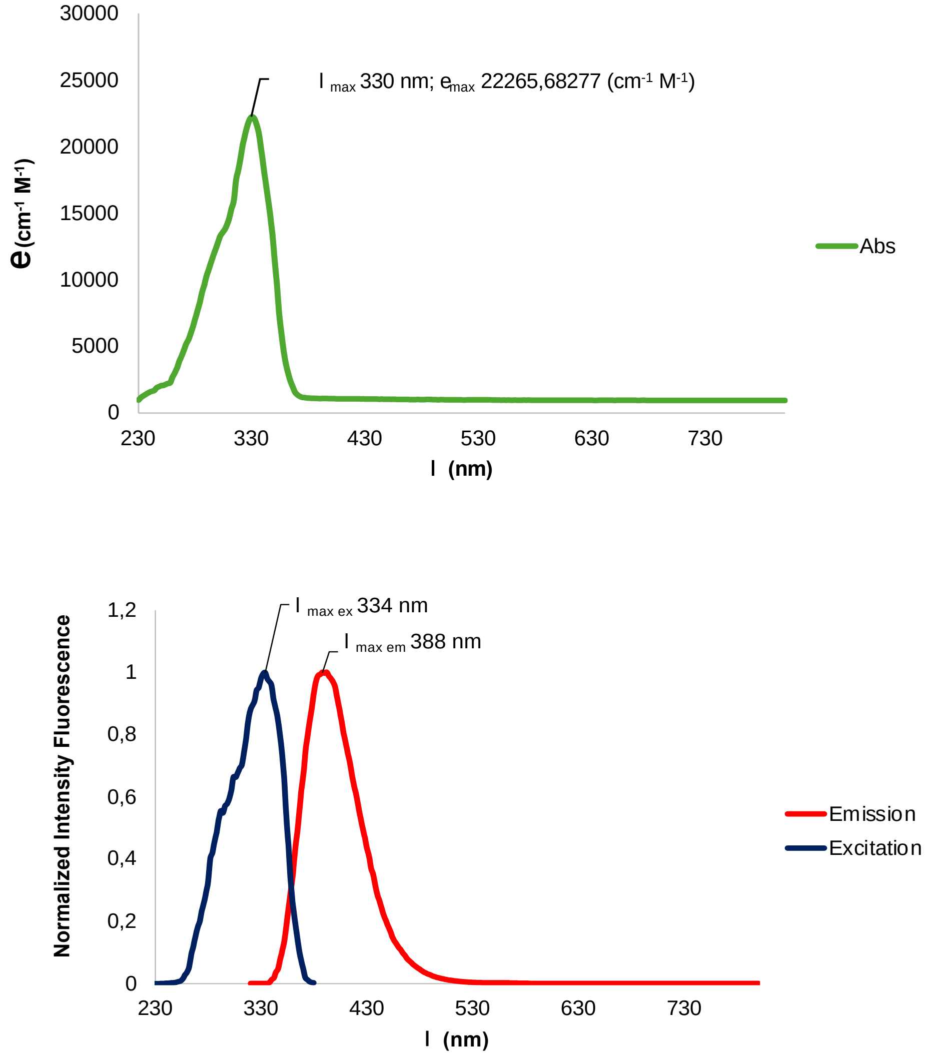

Figure S33. Absorbance and normalized fluorescence emission/excitation spectra of **S-4** 0.1 mM in DMF

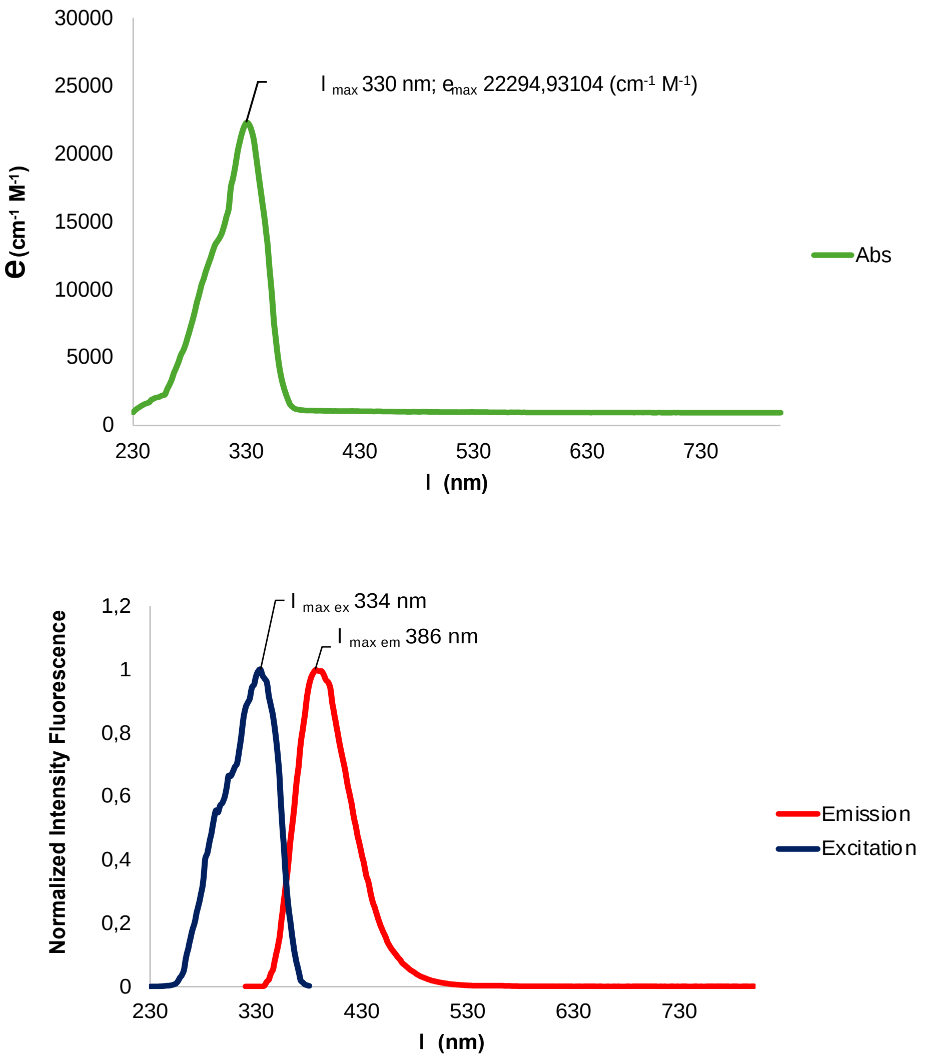

Figure S34. Absorbance and normalized fluorescence emission/excitation spectra of **S-5** 0.1 mM in DMF

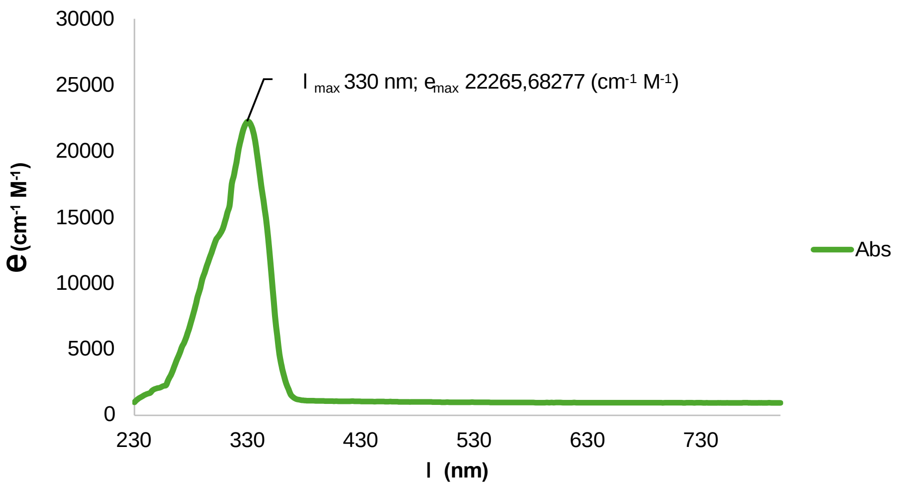

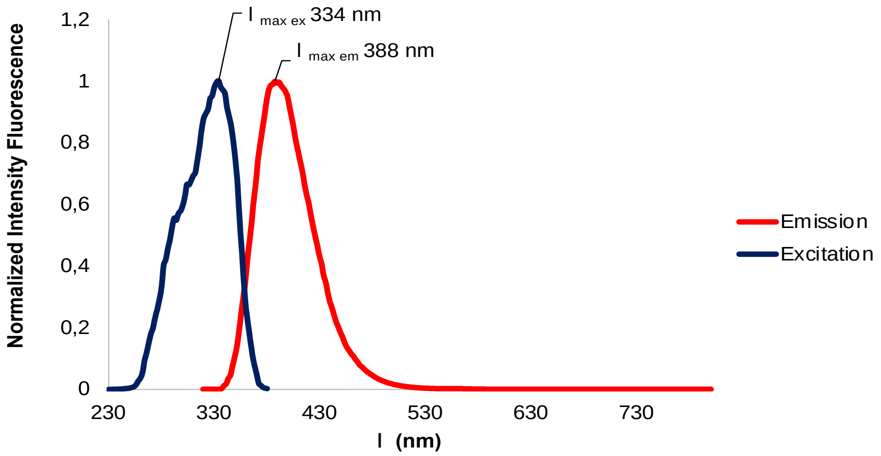

Figure S35. Absorbance and normalized fluorescence emission/excitation spectra of **S-6** 0.1 mM in DMF

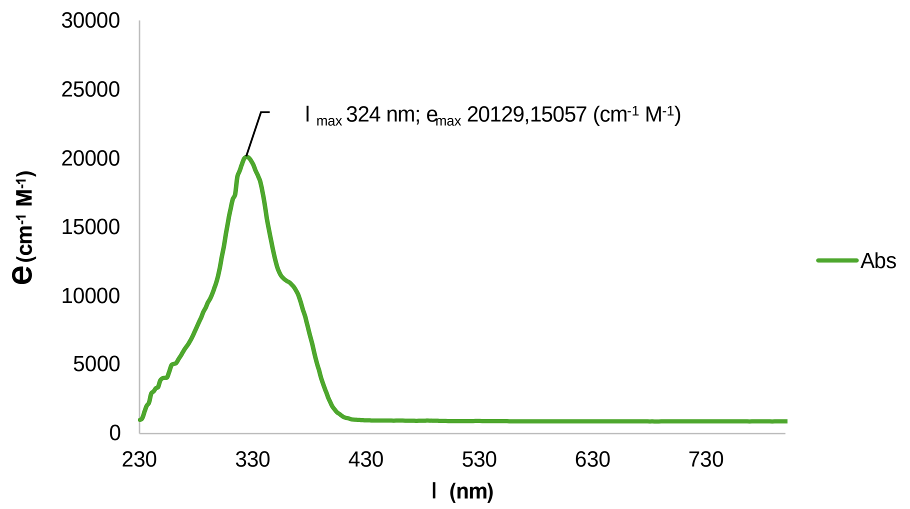

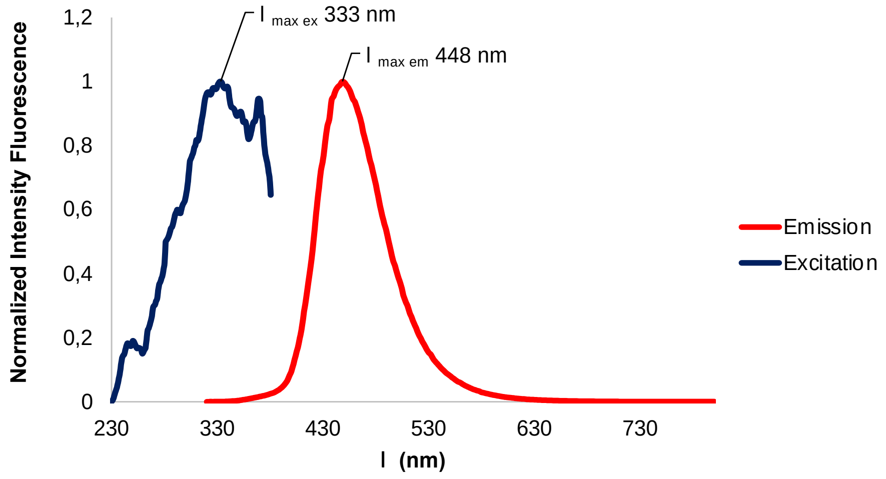

Figure S36. Absorbance and normalized fluorescence emission/excitation spectra of **4-MUB** 0.1 mM in PBS 1x (pH = 7.4)

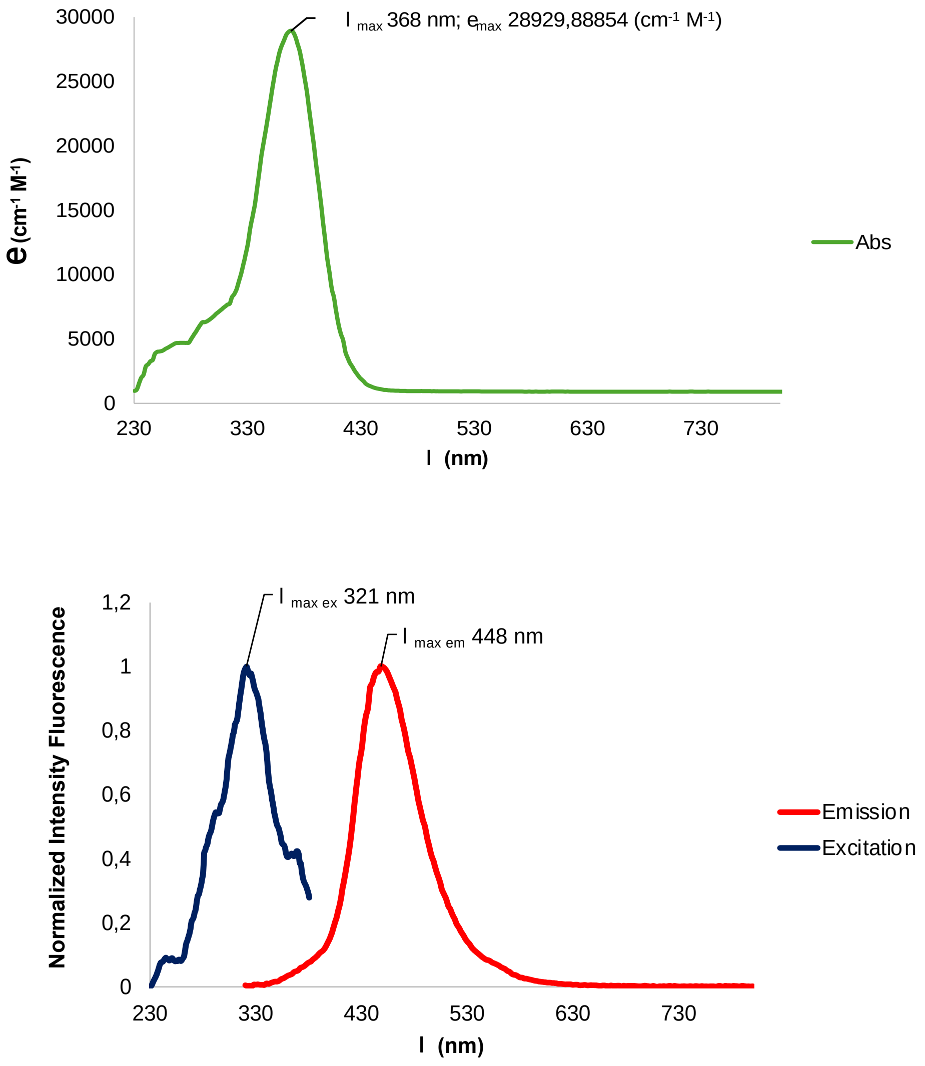

Figure S37. Absorbance and normalized fluorescence emission/excitation spectra of **7-MC** 0.1 mM + **DTT** 10 mM in PBS 1x (pH = 7.4)

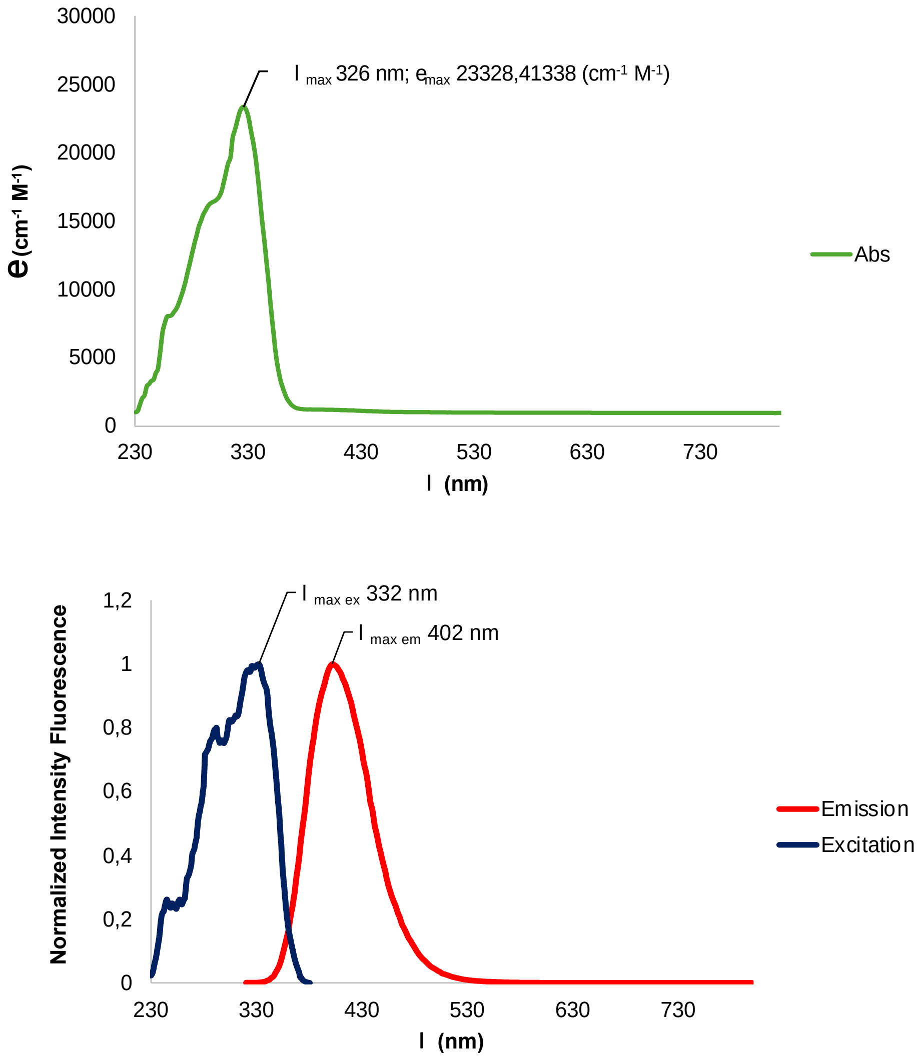

Figure S38. Absorbance and normalized fluorescence emission/excitation spectra of **S-1** 0.1 mM in PBS 1x (pH = 7.4)

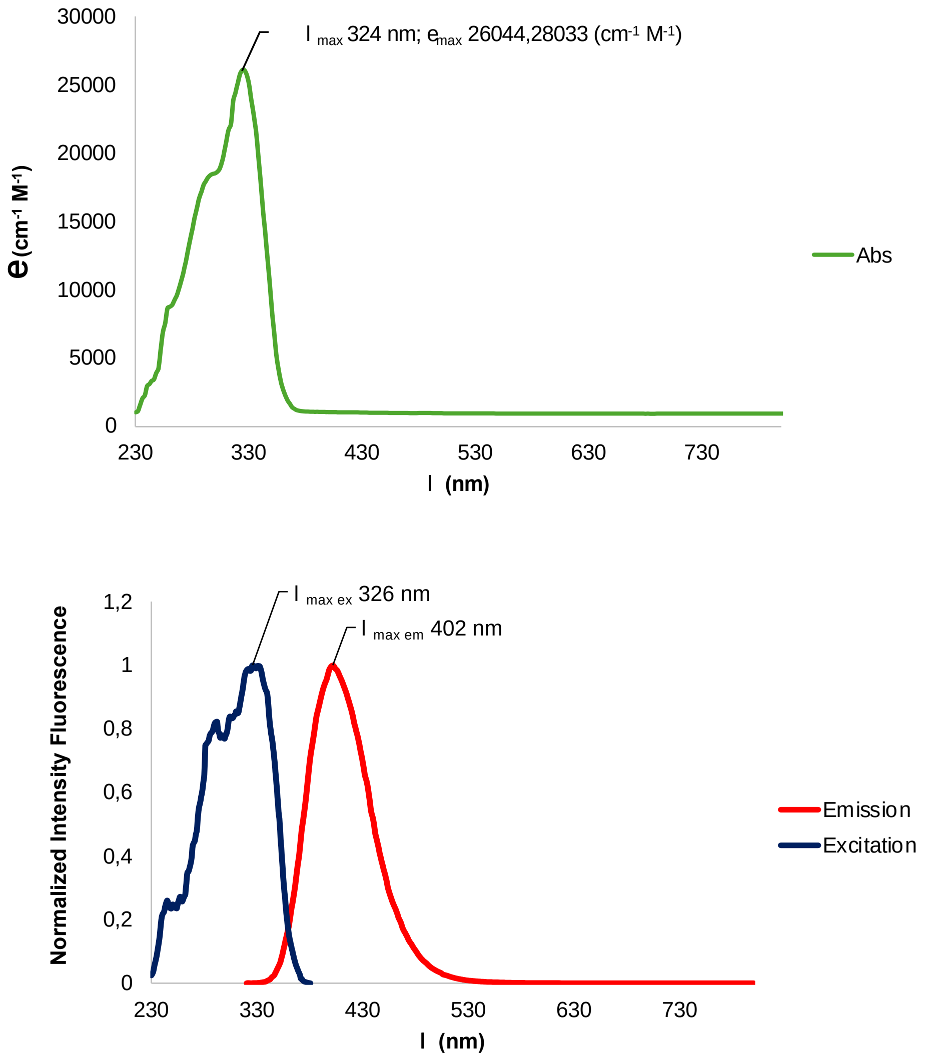

Figure S39. Absorbance and normalized fluorescence emission/excitation spectra of **S-2** 0.1 mM in PBS 1x (pH = 7.4)

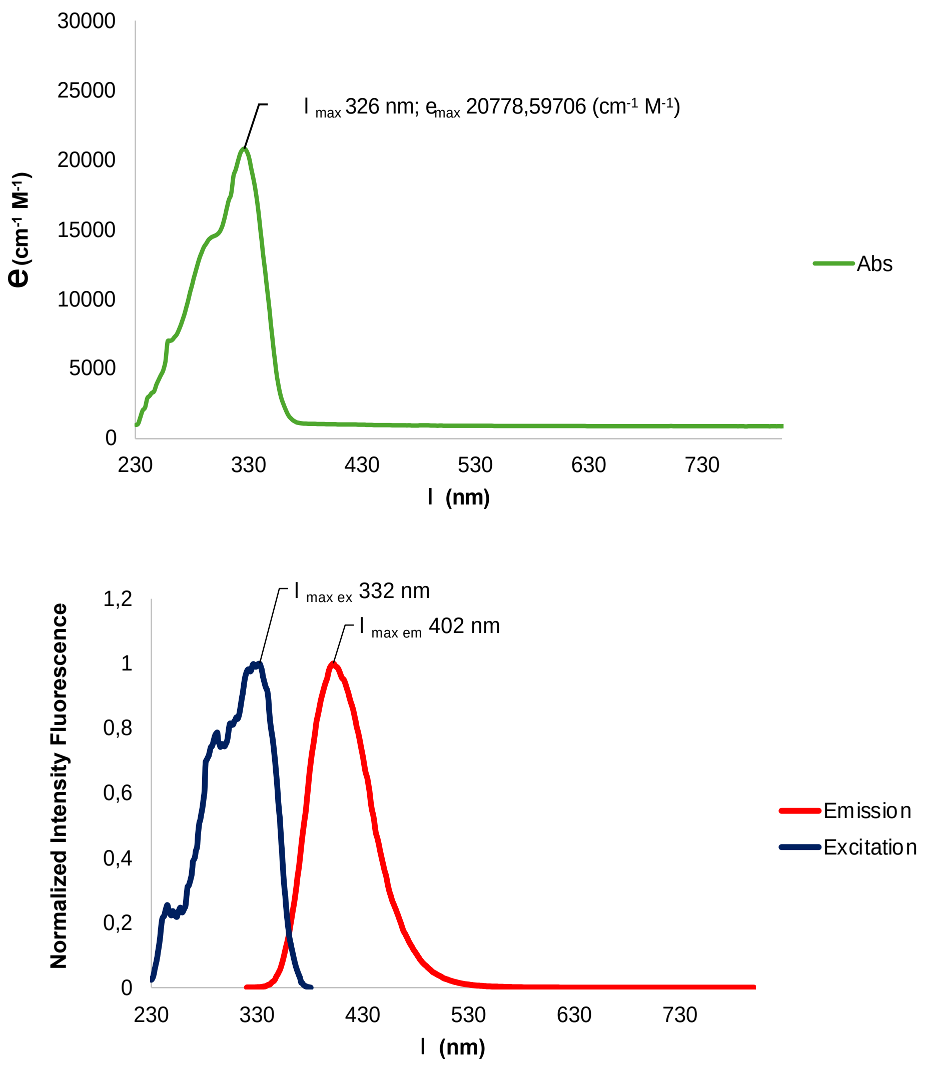

Figure S40. Absorbance and normalized fluorescence emission/excitation spectra of **S-3** 0.1 mM in PBS 1x (pH = 7.4)

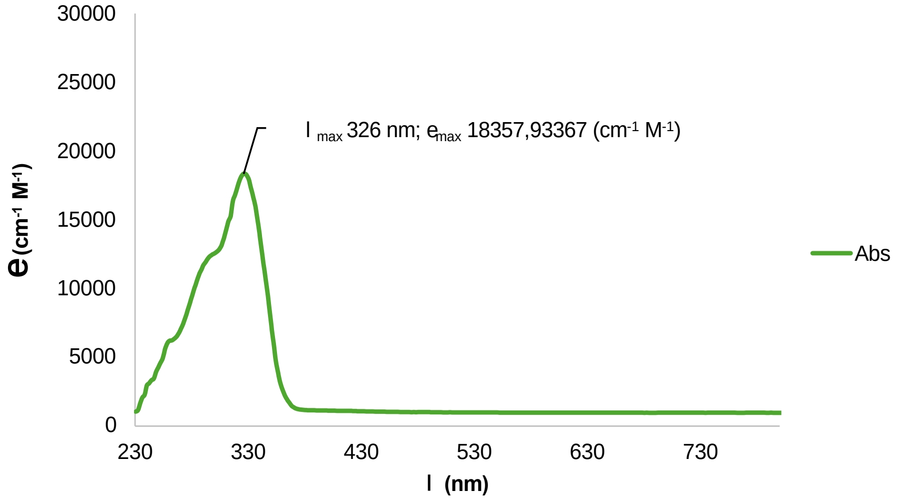

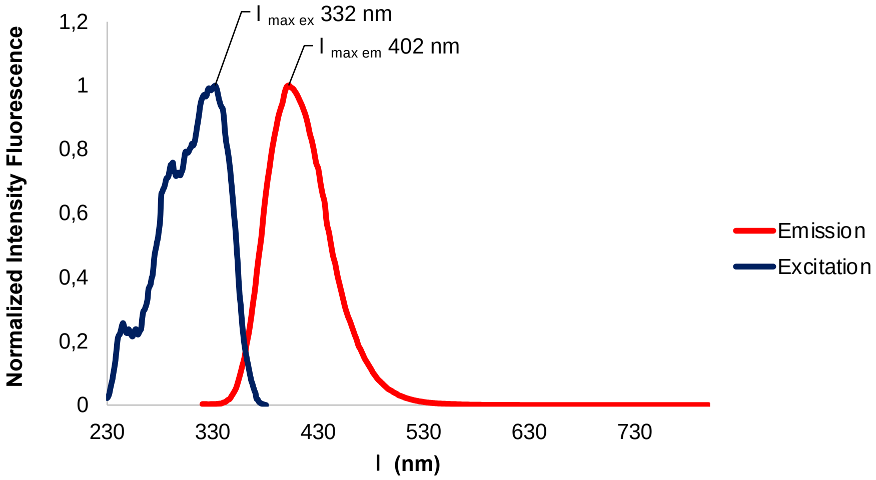

Figure S41. Absorbance and normalized fluorescence emission/excitation spectra of **S-4** 0.1 mM in PBS 1x (pH = 7.4)

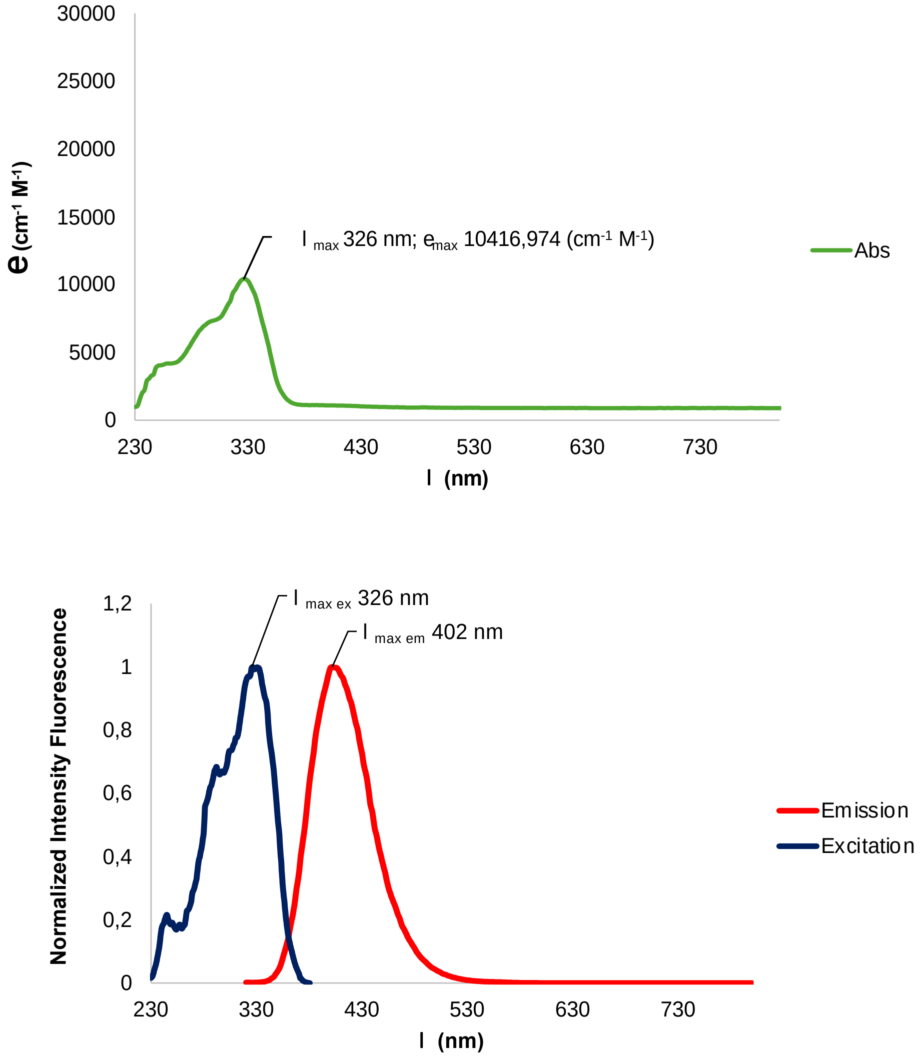

Figure S42. Absorbance and normalized fluorescence emission/excitation spectra of **S-5** 0.1 mM in PBS 1x (pH = 7.4)

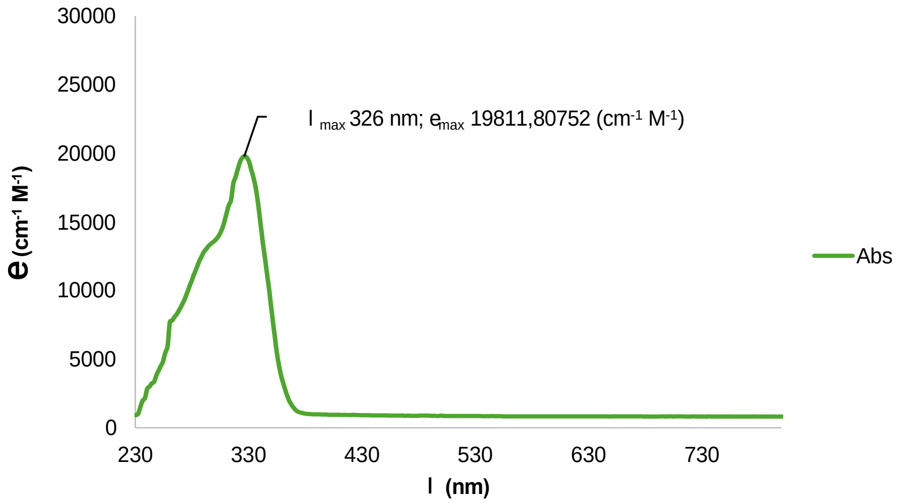

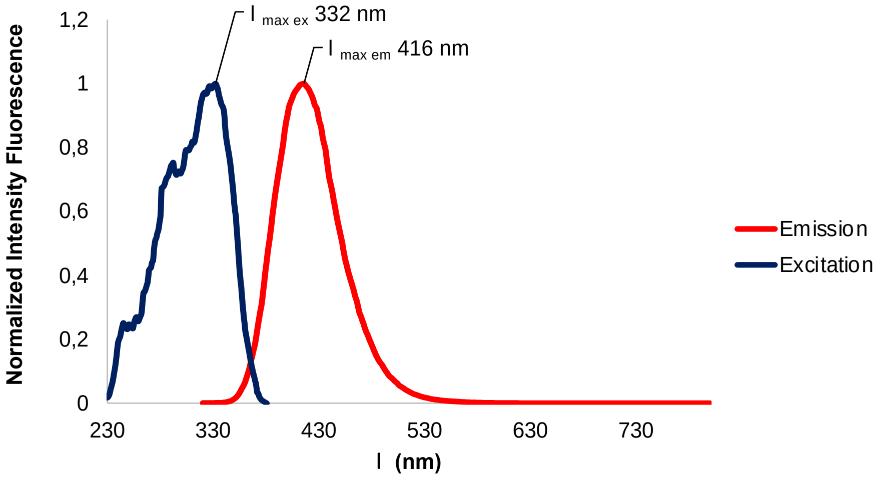

Figure S43. Absorbance and normalized fluorescence emission/excitation spectra of **S-6** 0.1 mM in PBS 1x (pH = 7.4)

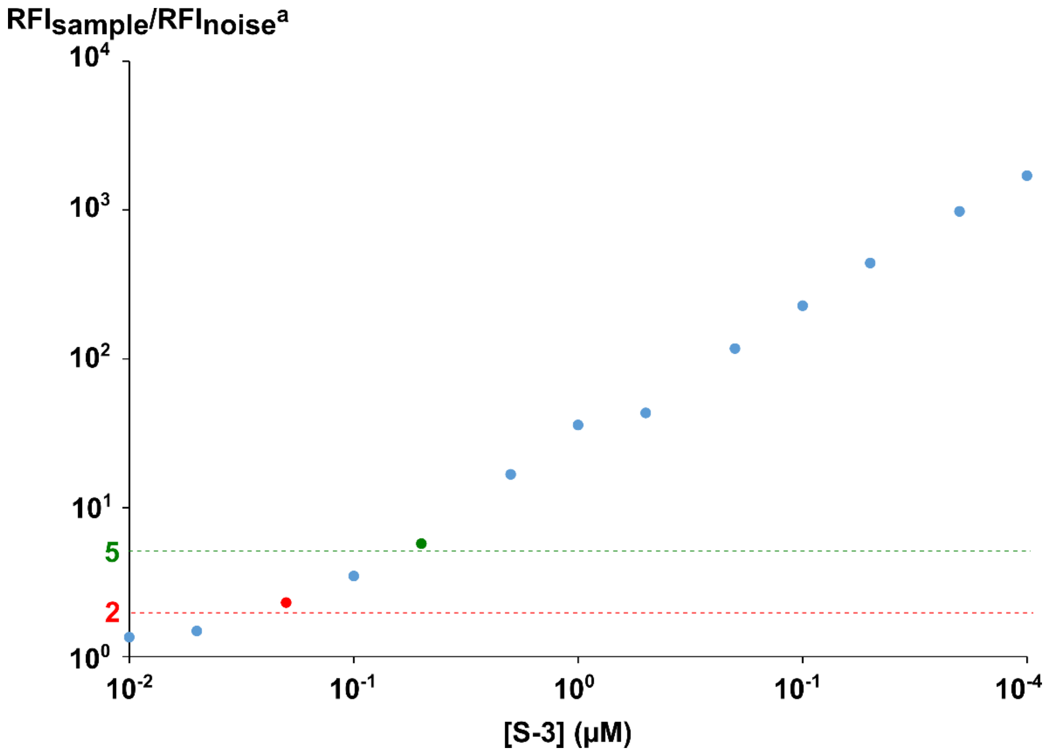

Figure S44. LOD & LOQ determination of **S-3** in PBS buffer. [a]RFI:Relative Fluorescence Intensity. The green (resp. red) dashed line correspond to the LOQ (resp. LOD). The minimum concentrations that gave signal/noise ratio above LOQ (resp. LOD) are indicated in the corresponding color, as were found to be 0.2 μM (resp 0.05 μM).
